# Leaf development regulates state transition capacity in trees

**DOI:** 10.1038/s41467-026-76469-5

**Published:** 2026-08-06

**Authors:** Chen Hu, Sanchali Nanda, Maria Dolores Pissolato, Maximiliano Cainzos, Tatyana Shutova, Stefan Jansson

**Affiliations:** 1 Umeå Plant Science Centre, Department of Plant Physiology, Umeå University, Umeå, Sweden; 2 Present Address: CSIR-Centre for Cellular and Molecular Biology, Hyderabad, India

**Keywords:** Plant sciences, Developmental biology, Photosynthesis

## Abstract

State transitions (ST) balance excitation energy between photosystem I and II. This process has been extensively studied in *Arabidopsis* but the regulation and physiological significance of ST in other angiosperms remain largely unknown. Here, we investigate ST in hybrid aspen and other tree species using physiological, biochemical, ultrastructural, and genetic approaches. We discover a pronounced canopy gradient in greenhouse-grown aspens, with young, upper leaves exhibiting substantially higher fluorescence-derived state-transition capacity (qT) than lower, older leaves. Seasonal monitoring of field-grown trees reveals a conserved developmental decline in qT across species. Reduced qT correlates with increased grana stacking and lower LHCII/PSII ratios, but not with LHCII phosphorylation, suggesting developmental remodeling of thylakoid architecture may influence the functional reorganization of PSII antenna connectivity during state transitions. Using the serine/threonine protein kinase (STN7) knockout mutant characterized outside *Arabidopsis*, we show that aspens lacking ST exhibit altered PSI/PSII ratios, reduced PSII operating efficiency in young leaves, and slower growth under greenhouse conditions with naturally variable light conditions but not under constant-light climate room condition. These findings indicate that ST is important for performance under dynamic light conditions, particularly in young leaves, and reveal a previously unrecognized developmental control of photosynthetic regulation.

## Abstract (web summary)

This study shows that young tree leaves are better able than older leaves to adjust to changing light conditions. By combining greenhouse, outdoor, and genetic studies, it reveals a developmental control of this process and its importance for growth under fluctuating light.

## Introduction

In plants, solar energy is captured by photosynthetic machinery to convert CO_2_ into organic molecules and release O_2_. Driven by light, photosystem II (PSII) oxidizes the water molecules and transfers the obtained electrons through the other photosynthetic complexes of electron transport chain, such as Cyt *b*_6_*f* and photosystem I (PSI), producing NADPH and establishing a transmembrane proton gradient essential for ATP synthesis and photoprotection. As PSII and PSI work in series and have distinct preferred wavelength absorbance their relative excitation levels must be dynamically balanced to sustain sufficient electron transport and prevent photodamage under fluctuating light.

State transition (ST) is a rapid short-term mechanism for this balance by reversibly adjusting absorption cross-sections of the two photosystems^1,2^. ST is regulated by the redox state of the plastoquinone pool^3–8^. Under PSII-light, the plastoquinone pool is reduced and the major light-harvesting complexes II (LHCII) are phosphorylated by the chloroplastic serine/threonine-protein kinase STN7. Phosphorylated LHCII undergoes dissociation from PSII and/or partial redistribution within the thylakoid membrane, including association with PSI-LHCI complexes, thereby contributing to the balancing of excitation energy between the photosystems^3,9–13^. This state is called state 2^14^; Reversely, under PSI-light, the plastoquinone pool is oxidized, LHCII is dephosphorylated by protein phosphatase 1 (PPH1/TAP38) and dissociates from PSI, restoring state 1^15^. While the amplitude of ST in higher plants (5-12%) is lower than in *Chlamydomonas* (60–80%)^5,6,16^, its physiological significance is evident since mutants defective in ST could show reduced growth and lower seed production^17,18^.

Extensive studies using the annual model species, *Arabidopsis*^3,9–12^, mainly grown under controlled (artificial) conditions, has been performed. However, the importance of ST in perennials such as trees, which dominate forest ecosystems, remains poorly understood. Trees can exhibit a strong vertical difference in leaf traits such as pigment composition and light use efficiency^19–22^. These differences are often attributed to light environment but also reflect developmental differentiation, raising the question of how ST is regulated across canopy and developmental contexts. To address this knowledge gap, we investigated ST in aspen (*Populus*), a tree model system, phylogenetically close to *Arabidopsis*, well suited for studying perennial growth and development^23,24^. Specifically, we quantified ST capacity across vertical leaf layers of greenhouse-grown hybrid aspen WT (T89, *P. tremula x tremuloides*, a genotype suitable for transformation) exposed to long-term dynamic light and also used CRISPR/Cas9 to generate *stn7* knockout mutants in the T89 background to functionally test the role of ST in trees. In parallel, we also tracked seasonal changes of ST in mature aspens (*P. tremula)* and three other tree species (*Prunus maackii*, *Sorbus aucuparia*, and *Betula pendula)* grown under natural field conditions. Through a combined physiological, biochemical, and ultrastructural approach, we demonstrate that ST in trees is strongly dependent on the leaf developmental stage, which has a stronger effect than light environment alone. Our study expands the understanding of dynamic photoregulation in plants grown in a natural environment. Moreover, as trees dominate global primary productivity and carbon cycling, understanding of how ST scales from molecular regulation to canopy architecture could provide insights into forest ecosystem functions and carbon balance.

## Results

### A vertical gradient of ST capacity in greenhouse-grown aspens

Aspens, including hybrid aspen WT (T89), have two different (heteroblastic) leaf forms and shoot growth habits. Grown in nature, overwintering buds formed in the autumn flush in the spring and pre-formed, rounded leaves with flat petioles developed in a short shoot^21^. Hence, in a mature tree grown in the field, all leaves are the same age. Young trees grown under optimal greenhouse/climate chamber conditions grow instead continuously and form long shoots and new leaves with a different shape, cordate, and with rounded petioles. This growth habit is maintained for years if shoots are continuously cut back. In nature, rapidly growing long shoots could also be formed, for example, when suckers are formed from roots or when a large fraction of the canopy is removed. In this study, we used this dual growth habit to, on one hand, compare leaves of different ages/positions in a tree and, on the other hand, follow one leaf cohort over time.

**Fig. 1:**
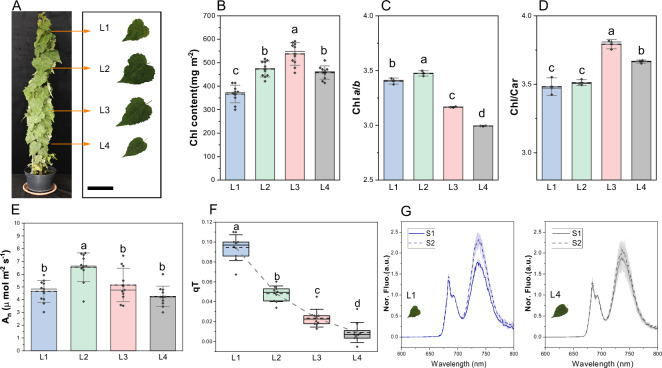
Vertical variation in state transitions capacity within greenhouse-grown aspen (WT (T89)). **A** Representative image tree of a T89 tree and leaves from different vertical layers (L1–L4). Scale bar, 10 cm. **B** Chlorophyll content (mg m^-2^) across leaf layers (*n* = 10, independent biological replicates (individual trees) per leaf layer). **C** Chlorophyll *a*/*b* ratio (*n* = 3, independent biological replicates per leaf layer). **D** Chlorophylls to carotenoids ratio (*n* = 3, independent biological replicates per leaf layer). **E** Net CO_2_ assimilation rate (A_n_, *n* = 11, independent biological replicates (individual trees) per leaf layer). **F** State transitions levels across different layers, quantified using Dual-PAM-100 and ChloroSpec chlorophyll fluorometer (*n* = 10, independent biological replicates (individual trees) per leaf layer). Data were presented as box plots, with the median indicated by the dashed lines, the lower and upper bounds of the box representing the 25th and 75th percentiles, respectively, and the whiskers representing the standard deviation. **G** 77 K chlorophyll fluorescence emission spectra from thylakoids of L1 (left) and L4 (right). Excitation wavelength, 475 nm. Shaded areas indicate s.d. (*n* = 3, independent biological replicates). Measurements were performed using 7-month-old-trees. The spectra were normalized at 692 nm. Data in **B**–**E** are presented as mean ± s.d. In **B**–**F**, different letters indicate significant differences between leaf layers based on two-sided one-way ANOVA followed by Tukey’s multiple-comparisons test (*P* < 0.05). Overall ANOVA results are: **B**, *F*(3,36) = 34.42, *P* < 0.0001; **C**, *F*(3,8) = 420.05, *P* < 0.0001; **D**, *F*(3,8) = 41.94, *P* < 0.0001; **E**, *F*(3,40) = 9.99, *P* < 0.0001; **F**, *F*(3,36) = 135.07, *P* < 0.0001. The complete ANOVA results and exact *P* values for all pairwise comparisons are provided in the Source Data.

In trees grown in greenhouse, we compared leaves vertically, from young top leaves to older bottom leaves. Leaves were divided into four layers (L1-L4). L2 and L3 leaves were larger than those in the youngest top (L1) and oldest bottom (L4) layers (Fig. 1A). L1 leaves had lowest chlorophyll content and L3 and L4 the highest (Fig. 1B) while upper leaves (L1 and L2) contained more carotenoids than lower (L3 and L4) (Fig. 1D). Pigment composition also varied: upper leaves (L1-L2) displayed higher Chl *a*/*b* ratios than lower leaves (L3 and L4), indicating variation in photosynthetic complexes across different layers (Fig. 1C). Although L1 leaves still displayed characteristics of developing leaves, they were photosynthetically highly active, CO_2_ fixation rates were comparable among layers, only L2 exhibited a higher assimilation rate than other layers (Fig. 1E).

ST capacity displayed a pronounced vertical gradient. L1 leaves exhibited the highest qT ( ~ 10%, Fig. 1F, Supplementary Fig. 2A, C), which declined progressively toward the bottom, reaching nearly undetectable levels in L4 (Fig. 1F, Supplementary Fig. 2B, D). 77 K fluorescence confirmed this trend: L1 thylakoids displayed a clear difference between state 1 and state 2, while L4 showed little change (Fig. 1G). Comparisons between trees with 4-months-, 7-months-, and 1-year-old shoots, grown under the same conditions, showed that the vertical gradient was consistent over time, though overall qT levels declined in all classes with age (4-month> 7-month>1 year, Supplementary Fig. 4), hence the capacity for qT was dependent both on leaf age and position in the canopy. NPQ values under HL were higher in L1 than in L4 (Supplementary Fig. 5A).

**Fig. 2:**
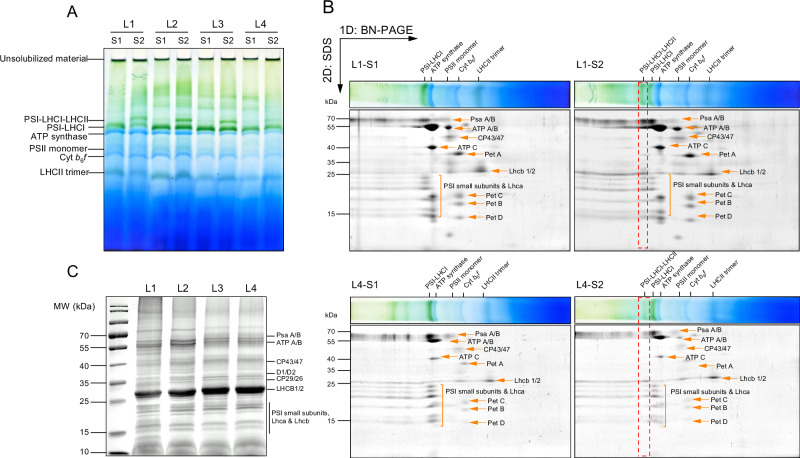
Layer-dependent biochemical organization of photosynthetic complexes in aspen. Blue Native PAGE (BN-PAGE) of thylakoids isolated from the different leaf layers (L1–L4) in state 1 and state 2. Thylakoids were solubilized with 2% digitonin. Equal amounts of chlorophyll were loaded in each lane. **B** Second-dimension SDS-PAGE analysis of BN-PAGE complexes from leaf layers L1 (top panel) and L4 (lower panel). **C** Coomassie-stained SDS-PAGE of thylakoid proteins across different leaf layers. The experiment shown in **A**–**C** was repeated independently 3 times with similar results. The relevant raw images were provided in the Source Data.

To validate the physiological observations, we used BN-PAGE and SDS-PAGE to compare photosynthetic complexes in L1 and L4 leaves. Under state 2, L1 thylakoids displayed PSI-LHCI-LHCII complexes, which were absent in state 1 (Fig. 2A). This complex was also present, although at a lower level, in L4 in state 2 (Fig. 2A) leaves had negligible measured qT values (Fig. 1F). This suggests that PSI association and fluorescence-based qT measurements report partially distinct aspects of ST-related antenna reorganization. Digitonin solubilization efficiency was lower in L4 thylakoids than in L1 (24 ± 4% and 35 ± 1%, respectively, Supplementary Fig. 6C). Two-dimensional SDS-PAGE revealed comparable overall protein patterns with *Arabidopsis* (Fig. 2B, Supplementary Fig. 6A, and Supplementary Fig. 6B). Coomassie-stained denaturing SDS-PAGE gels showed similar overall protein profiles across the layers, with only minor visible differences in the intensities of some individual proteins (Fig. 2C).

### Chloroplast ultrastructure differs between leaf layers

**Fig. 3:**
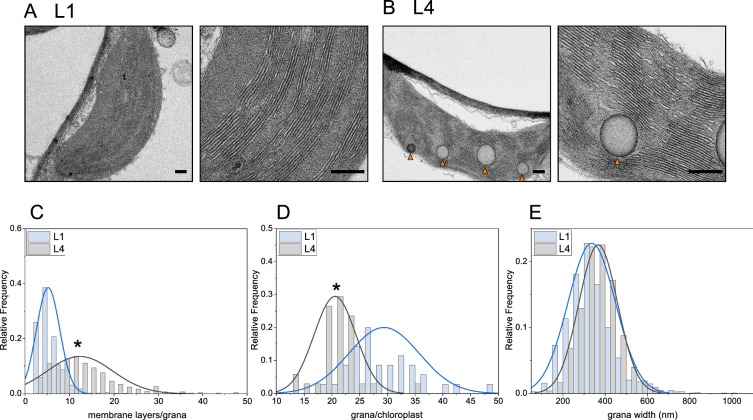
Ultrastructural differences in chloroplasts between top (L1) and bottom (L4) layers of aspen leaves. **A**, **B** Representative transmission electron micrographs of chloroplasts from L1 (**A**) and L4 (**B**) leaves in state 1. Plastoglobuli are indicated by orange triangles in the L4 chloroplast. Scale bar, 300 nm. **C**–**E** Quantitative analysis of chloroplast ultrastructure. **C** number of membrane layers per grana (*n* = 1002 grana measured in L1 and *n* = 459 grana measured in L4), **D** number of grana per chloroplast (*n* = 35 chloroplasts in L1 and *n* = 34 chloroplasts in L4), **E** grana width (*n* = 1024 grana in L1 and *n* = 728 grana in L4). Histograms were fitted with a Gaussian function. For each leaf layer, five leaves were collected from each of three independent trees and pooled by tree before sample preparation, resulting in three independent biological samples per leaf layer. Statistical comparisons were performed using two-sided Welch’s t-tests. For **C**, *t* (511.02) = − 19.99, *P* < 0.0001; for **D**, *t* (55.57) = 7.05, *P* < 0.0001; and for **E**, *t* (1716.50) = − 6.26, *P* < 0.0001. Asterisks indicate statistically significant differences between leaf layers (*P* < 0.05). The corresponding test statistics and exact *P* values are provided in the Source Data.

Next, we used Transmission Electron Microscopy (TEM) to study thylakoid architecture in different leaf layers and found very pronounced differences (Fig. 3A-B). The grana stacks in L4 leaves contained much more layers of thylakoids per granum than L1 leaves (L1); almost no stacks were over 10 layers in L1 whereas ca. half of the L4 grana stacks had over 10 layers, some over 40 (Fig. 3C). On the other hand, the number of grana stacks was much higher in L1 leaves (Fig. 3D), while grana diameters were similar across layers (Fig. 3E). In terms of ST, both L1 and L4 reduced stacking upon transition to state 2, as expected. However, the differences induced by changing light conditions were much smaller than the differences between leaf layers (Supplementary Fig. 7). Plastoglobuli were observed in chloroplasts from all developmental stages, but they became larger and more abundant in L4 leaves (Fig. 3B, Supplementary Fig. 7F and G). Plastoglobuli are constitutive components of plastids throughout development but are known to increase in size and abundance during leaf aging and senescence. Plastoglobuli were less abundant in L4 than in fully senescing aspen leaves^25^, consistent with chlorophyll levels and CO_2_ fixation rates measurements showing that L4 leaves, although relatively old, were not yet senescent but still photosynthetically highly active.

### Seasonal dynamics in outdoor *Populus* and other trees species

**Fig. 4:**
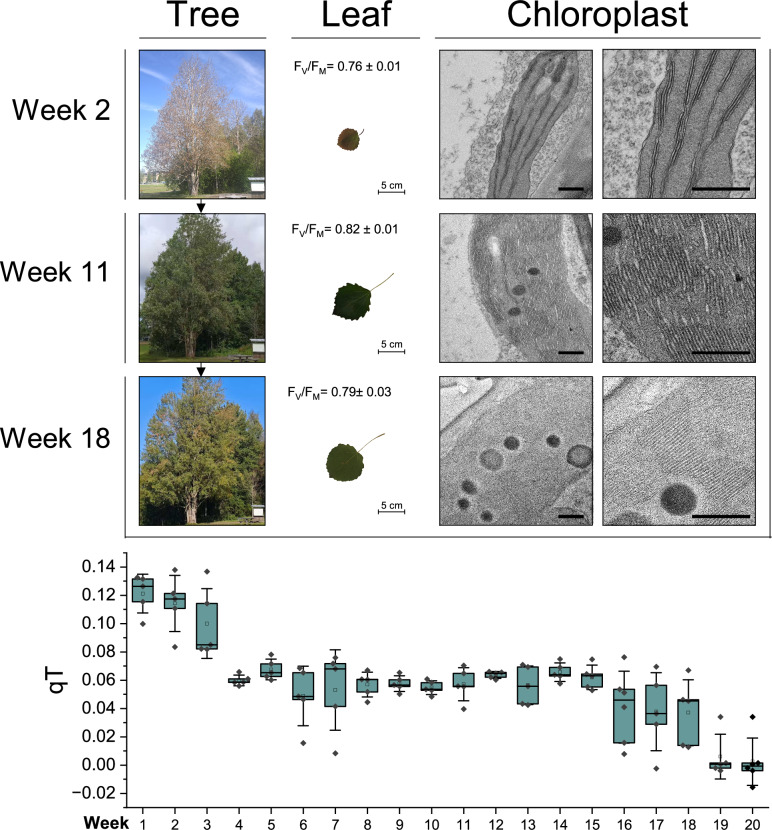
Seasonal dynamics of state transitions capacity in outdoor aspen trees. Left and central panels: representative photographs of the *P. tremula* tree in Umeå (N63°49’ E20°18’), Sweden, taken from late spring (Week 1, 28th May) to late autumn (Week 20, 9th Oct) 2025, alongside representative images of the leaves collected at different time points. Right panel: representative transmission electron micrographs of chloroplasts sampled in early June (Week 2), early August (Week 11) and late September (Week 18); scale bars = 500 nm. Bottom panel: seasonal changes in state transition capacity plotted over time. Each data point represents the qT value measured from an individual leaf. At each time point, one leaf was collected from each of five independent trees (*n* = 5 trees per time point). Data are presented as individual measurements. The relevant data were provided in the Source data.

To obtain better resolved data on ST in leaves of different ages we measured ST weekly over the whole growing season, from leaf emergence to leaf abscission in aspens in a natural stand on the Umeå university campus (Fig. 4). This would show to what extent ST were expressed in aspen trees under natural conditions and allow for separation, compared to the greenhouse grown aspens analyzed above, of the effects of leaf age and canopy position. Although measurements from trees in nature are inevitably less reproducible, the results were similar to those from the greenhouse. High levels of ST (over 10%) were recorded in young pale, still developing leaves during the first two weeks, and values kept stable (5–6%) throughout the summer. When leaves entered senescence in September, the levels dropped even further and highly senescent leaves lacked measurable ST. The development of the thylakoid ultrastructure also matched the results from the greenhouse (Fig. 4, Supplementary Fig. 8A). Notably, NPQ under HL behaved somewhat similarly: higher levels in younger leaves but lower in older leaves (Supplementary Fig. 5B). Immunoblot analysis further revealed a gradual decrease in the relative LHCII/PSII ratio during the seasonal progression (Supplementary Fig. 8B), indicating that seasonal remodeling of photosystem stoichiometry accompanies the reduction in qT.

We also decided to quantify qT over time in leaves of other tree species grown under natural conditions. Very similar trends were observed: *Prunus maackii* dropped from ~6% to <1%, in *Sorbus aucuparia* dropped from ~7% to <1% and declined from ~8% to ~1% in *Betula pendula* from mid-summer to autumn (Supplementary Fig. 9). Obviously, this developmentally programmed regulation of ST capacity is a conserved feature.

### Aspen *stn7* mutants behaved like *Arabidopsis stn7*

**Fig. 5:**
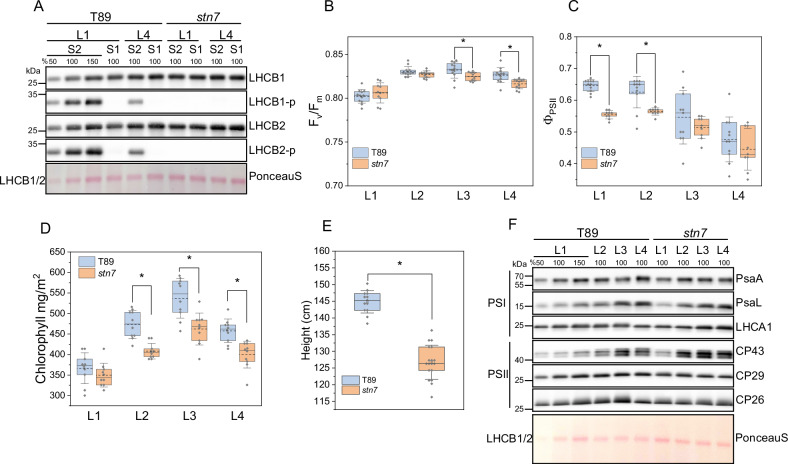
Physiological and molecular characterization of stn7 mutant in aspen WT (T89). Representative immunoblot analysis of LHCII phosphorylation in L1 and L4 of T89 and *stn7*. Samples were equalized based on chlorophyll content prior to loading. **B** Maximum quantum yield of PSII photochemistry (F_v_/F_m_, *n* = 12 for T89 and *n* = 10 for *stn7*, independent biological replicates) and (**C**) steady-state PSII light use efficiency (at 150 μmol photons/m^2^/s, *n* = 11 for T89 and *n* = 9 for *stn7*, independent biological replicates). **D** Comparison of chlorophyll content between WT (T89) (same as in Fig. 1B) and *stn7* across layers (*n* = 10 independent biological replicates per genotype and leaf layer, with each replicate derived from an individual tree). **E** Tree height after 4 months of growth in greenhouse (*n* = 13 in T89, *n* = 18 in *stn7*, independent biological replicates). **F** Representatives immunoblot analysis of protein composition in L1 and L4. The L1 WT(T89) was included as a reference between blots. Ponceau S-stained membranes are shown below to indicate protein transfer efficiency. The corresponding Coomassie-stained gels are shown in Supplementary Fig. 10C. The experiment was repeated independently 3 times with similar results. Data in panels B-E are presented as box plots, with the median indicated by the dashed line, the lower and upper bounds of the box representing the 25th and 75th percentiles, respectively, and the whiskers representing the standard deviation. Statistical comparisons between T89 and stn7 were performed using two-sided one-way ANOVA separately for each leaf layer. For **B**, *F* (1, 20) = 0.90, *P* = 0.355 for L1; *F* (1,20) = 2.29, *P* = 0.146 for L2; *F* (1,20) = 5.19, *P* = 0.0338 for L3; and *F* (1,20) = 10.94, *P* = 0.00335 for L4. For **C**, *F* (1,18) = 148.29, *P* < 0.0001 for L1; *F* (1,18) = 12.91, *P* = 0.00208 for L2; *F* (1,18) = 1.13, *P* = 0.302 for L3; and *F* (1,18) = 1.00, *P* = 0.331 for L4. For **D**, *F* (1,18) = 1.28, *P* = 0.272 for L1; *F* (1,18) = 28.24, *P* < 0.0001 for L2; *F* (1,18) = 14.66, *P* = 0.00123 for L3; and *F* (1,18) = 17.91, *P* = 0.000501 for L4. For **E**, *F* (1,29) = 124.86, *P* < 0.0001. The corresponding overall test statistics and exact *P* values are provided in the Source Data.

To directly test ST function in aspen we used genome editing to generate two independent *stn7* mutant lines. Both lines showed in all aspects indistinguishable phenotypes, therefore, we will in the following show data from both lines combined. As expected, *stn7* mutants lacked LHCII phosphorylation and PSI-LHCI-LHCII complexes formation upon induction of state 2 (Fig. 5A, Fig.S10A). Interestingly, L4 leaves of T89 (WT) still retained ~60% LHCII phosphorylation level relative to L1 (Fig. 5A, Supplementary Fig. 10B) and the relative abundance of PSI-LHCI-LHCII complexes were also reduced in L4 leaves (Fig. 2A and Supplementary Table 2), despite their level of ST was ca. 10-fold lower (Fig. 1F).

Functionally, maximum PSII quantum yield (F_v_/F_m_) was comparable between WT (T89) and *stn7* in top (L1 and L2) but was in *stn7* slightly lower in L3 and L4 (Fig. 5B). By contrast, steady-state PSII light use efficiency (Φ_PSII_) under growth light was lower in L1- L2 of *stn7* relative to WT (T89), but not in L3 and L4 (Fig. 5C). Chlorophyll content across layers was somewhat more uniform in *stn7* leaves (Fig. 5D), but in all layers except L1, *stn7* contained less chlorophyll than in WT (T89) (Fig. 5D).

Immunoblotting revealed layer-dependent, but only minor genotype-dependent differences in PSI/PSII and protein stoichiometries. PSI core proteins (PsaA and PsaL) levels in L4 were higher than in L1 in both genotypes (Fig. 5F), but PSI/PSII (PsaA/CP43) protein ratio was lower in L4 (Supplementary Fig. 10B). This ratio was also lower in *stn7* than in the corresponding layer of WT (T89). Notably, LHCII/PSII protein ratio was lower in L4 in both genotypes, indicating smaller PSII antenna in bottom leaves (Supplementary Fig. 10B). Minor antenna proteins (Lhca1, CP29, and CP26) showed comparable or slightly reduced levels in L4 relative to L1 but did not obviously differ between *stn7* and WT (T89) (Fig. 5F).

When grown in the greenhouse, *stn7* grew slower than WT (T89); after 4 months, the heights of *stn7* trees were ca. 10% lower (Fig. 5E). This prompted us to analyze growth in our phenotyping facility, where conditions are constant and also optimal for growth, and growth rate can be monitored with high precision. Here, the growth rates of *stn7* and WT (T89) were not different (Supplementary Fig. 10D).

### qT responses to a far-red-enriched canopy shading environment

To evaluate whether shading could explain the reduced qT observed in lower aspen leaves, we performed an additional shading experiment in which short aspens were grown beneath a dense canopy formed by taller aspens (Supplementary Fig. 11A). The shaded plants experienced a strongly far-red-enriched light environment (L4, Supplementary Fig. 11B) compared to control plants. However, after 12 days of shading treatment, qT values in both upper and lower leaves didn’t change compared to those measured in unshaded control plants (Supplementary Fig. 11C). Thus, a far-red-enriched canopy environment for 12 days did not reduce qT in mature leaves.

### Developmental variation in qT across the *Arabidopsis* rosette

To examine whether developmental changes in qT also occur in *Arabidopsis*, we measured qT using the SpeedZen chlorophyll fluorescence imaging system (Supplementary Fig. 12). Initial measurements of intact rosettes showed that qT was highest in younger leaves and tended to decrease in older leaves. Because leaf curvature, overlap, and leaf angle may cause distortions in fluorescence measurements, leaves were subsequently detached and grouped into three developmental classes (young, mature and old) for analysis on a common flat platform. Young and mature leaves displayed similar qT values, whereas the oldest leaves exhibited lower qT (Supplementary Fig. 12). The developmental differences in *Arabidopsis* were considerably smaller than those observed between L1 and L4 aspen leaves.

## Discussion

State transition is considered to be an important regulatory process for rebalancing excitation between PSI and PSII^1,2^. However, there are knowledge gaps, partially because virtually all previous studies^3,9–12^ have studied ST in *Arabidopsis* or algae grown under lab conditions. Because of this several interesting questions remain, at least in part, unanswered: How do ST operate in other angiosperms, how important are ST under natural conditions and what is the relationship between ST and thylakoid ultrastructure? We have addressed them by analyzing ST in aspen, including studies under various growth conditions, including growth chambers and natural (outdoor), comparing leaves of different positions and ages and generating mutants in the key protein (STN7).

### Developmental regulation of qT

Growth chamber grown *Arabidopsis* and indoor grown aspen (WT (T89)) show similar ST induction; also the aspens lacking STN7 behave quite similarly as *Arabidopsis stn7*; they lack measurable ST and phosphorylation of Lhcb1/Lhcb2, affecting photosynthetic function, for example displaying reduced Φ_PSII_ (Fig. 5C).

In greenhouse-grown trees, we observed a steep gradient in qT: young, upper-canopy leaves displayed high qT while old leaves had little or no qT. Outdoor seasonal observations (confirmed by studies on additional tree species) showed that qT capacity was quite high right after leaf emergence, lower throughout the summer and was decreasing or disappearing as leaves entered senescence.

Why is qT much higher in young, greenhouse grown aspen upper leaves? It could be advantageous for upper, younger leaves to have a higher capacity for ST and NPQ when their chloroplasts are yet not fully developed to preserve Φ_PSII_ and prevent transient photodamage, and they simply do not “know” yet to what light environment they should be optimized for; if they should become “sun leaves” or “shade leaves”. Consistent with this idea, *stn7* knockout trees lacked ST, displayed reduced Φ_PSII_ in upper leaves, and grew less well under fluctuating light but not under constant light, supporting the importance of ST for canopy photoregulation and whole plant performance in dynamic light.

As leaves mature (after a few weeks in the case of aspen), chloroplasts have been structurally optimized in terms of light harvesting, stacking, Calvin cycle capacity, etc. and reduce their regulatory flexibility. Our data suggest that qT capacity keeps up until the dismantling of chloroplasts during senescence, causing its decrease. Older leaves in the greenhouse experiment indeed displayed some features resembling senescence (more plastoglobuli and lower Chl *a*/*b* ratio)^26–29^. However, L4 leaves maintained high F_v_/F_m_, chlorophyll content, and CO_2_ assimilation rate, so differences between L4 and younger leaves could potentially also be explained by differences in light conditions. Bottom leaves are experiencing shading environment enriched with far-red light which can enhance grana stacking^30,31^ and could therefore potentially suppress ST and alter leaf developmental trajectories^32,33^. This makes it difficult to separate effects arising from direct photophysical changes from light-induced developmental reprogramming. However, the additional shading experiment where lower canopy leaves experience a far-red-enriched environment (Supplementary Fig. 11) showed that 12 days of shading did not reproduce the strong reduction in qT observed in mature lower leaves. Hence, differences in light environment alone cannot fully explain the developmental gradient in qT.

Long-term state 1 conditions may additionally contribute to reduced ST capacity through altered STN7 abundance, as previously reported in *Arabidopsis*^34^. In our hands, a commercially available *Arabidopsis* STN7 antibody was not able to detect aspen STN7, so alternative approaches will be required to better evaluate the role of STN7 abundance in regulating ST capacity during leaf development. Seasonal temperature changes may also contribute to the regulation of qT in outdoor trees. In particular, seasonal remodeling of photosystem stoichiometry may contribute to the observed reduction in qT (Supplementary Fig. 8). Such changes could alter the effective PSII antenna size and thereby affect fluorescence-based qT measurements. However, qT did not follow the seasonal temperature trend (Supplementary Fig. 9D). suggesting that reduction of in qT in the autumn cannot be explained by decreasing temperatures. Notably, we also found a similar, although weaker developmental qT decline in *Arabidopsis* rosette leaves (Supplementary Fig. 12), suggesting that developmental down-regulation of ST capacity is a general feature in angiosperms.

### Potential mechanisms underlying reduced qT in mature leaves

What are the mechanistic reasons underlying this reduction in qT? As the fluorescence signal at room temperature is dominated by PSII, currently available qT protocols primarily reflect changes in the effective PSII antenna cross-section rather than directly measuring large-scale physical redistribution of LHCII within the thylakoid membrane. We therefore first hypothesized that the reduced qT in older leaves could partly result from lower relative antenna availability. Consistent with this idea, both greenhouse-grown and outdoor mature leaves displayed lower LHCII/PSII ratios compared to younger leaves, and seasonal immunoblot analysis further revealed a progressive decline in the relative LHCII/PSII ratio during leaf aging (Supplementary Fig. 8 and Supplementary Fig. 10). However, antenna size alone is unlikely to fully explain the observed qT decline. In older leaves, substantial LHCII phosphorylation (60% of WT (T89)) was still detected, whereas chlorophyll fluorescence measurements showed only little qT. This suggests that LHCII abundance, phosphorylation and fluorescence-derived qT are not linearly coupled and that additional structural constraints may limit changes in effective PSII antenna size even when LHCII phosphorylation is maintained.

One possible explanation is the altered architecture of the thylakoid membrane. Lower/older leaves displayed highly stacked grana, which may stabilize PSII-LHCII connectivity and reduce the flexibility of antenna reorganization in qT measurements. Such developmental remodeling of chloroplast ultrastructure and thylakoid organization, including changes in grana architecture during leaf maturation, has previously been described in higher plants^29,35,36^. In addition, previous studies in *Arabidopsis* have shown that state transitions are associated with dynamic remodeling of thylakoid architecture, including changes in grana organization and membrane stacking^37–39^. We also detected layer-dependent differences in the relative abundance of several PSII subunits; such compositional differences could influence local photosystem organization and the LHCII functional associations. Interestingly, unicellular algae such as *Chlamydomonas*, which lack extensive grana stacking, exhibit much larger ST capacity^40^, consistent with the idea that thylakoid architecture strongly influences the dynamic range of antenna reorganization.

Notably, PSI-LHCI-LHCII assemblies were still detectable in older leaves under state 2 conditions despite their negligible qT values. This suggests that PSI-associated LHCII complexes and fluorescence-derived qT measurements reflect partially distinct aspects of ST-related antenna reorganization. This is consistent with recent models proposing that ST involve dynamic reorganization and partial redistribution of LHCII within the thylakoid membrane rather than complete long-distance migration between grana and stroma lamellae^13^.

### The role of state transitions in dynamic light environments

Finally, how important is ST for plants? Aspen mutants lacking STN7 grew perfectly fine under optimal, constant conditions in our phenotyping facility. However, under greenhouse conditions (with fluctuating light), *stn7* mutants grew slower than WT, and a small but measurable reduction in seed production of *Arabidopsis stn7* mutants grown in the field has at least some years also been measured^18^. This suggests that ST balancing excitation of PSI an PII fluctuating conditions is important. The altered PSI/PSII stoichiometry observed in *stn7* may also lead to long-term redox-driven adjustments^41–43^, but they may be insufficient to restore full growth performance. As it has been suggested that the STN7 kinase also regulates thylakoid remodeling and photosynthetic complex stability through phosphorylation of CP29^29^ in addition to regulating ST, some of the observed phenotypes in *stn7* aspens may arise from other effects. Ongoing long-term field experiments with our aspen lines may also shed additional light on the importance of STN7.

In summary, our results reveal that state transition capacity in trees is strongly linked to leaf development, with young leaves exhibiting high photoregulatory flexibility that declines as leaves mature. This decline occurs despite sustained LHCII phosphorylation, suggesting that the structural maturation of the thylakoid membrane may constrain the flexibility of antenna reorganization reflected in fluorescence-derived qT measurements. By generating the STN7 knockout mutant in a tree and quantifying its impact on light-use efficiency and growth under fluctuating conditions, we provide direct evidence that dynamic photoregulation is tightly integrated with leaf ontogeny. This means that, at least in long-lived plants, short-term photosynthetic regulation is closely linked to the developmental program of leaves, linking cellular architecture to leaf photoregulatory function. We also would like to highlight the fact that studies using other plant species, grown under natural conditions, can give insights about photosynthesis that complement knowledge obtained from *Arabidopsis* plants grown under lab conditions.

## Methods

### Plant growth

Hybrid aspen (*P. tremula x tremuloides*, T89, WT) and *stn7* mutant lines (see below) were grown in a glass greenhouse under dynamic light (natural sunlight plus supplemental white lamps; light profiles in Supplementary Fig. 1) or in the UPSC phenotyping platform (Sweden) under constant light. In both conditions, photoperiod was 18 h light / 6 h dark. Day/night temperature was 23 °C /18 °C with 70% relative humidity (RH). In the greenhouse, lamps automatically adjusted to achieve a minimum light intensity of ~150 μmol photons m^-2^ s^-1^during the photoperiod. Plants were grown for three distinct time periods: 4 months, 7 months, or 12 months. The 7-month- and 1-year-old trees were generated by repeated regrowth after cutting the main stem, allowing new shoots and canopies to develop from the remaining stem and root system. Leaf layers (L1-L4) were defined according to their relative vertical positions within the newly formed canopy, from the uppermost fully expanded leaves (L1) to the lowermost mature leaves (L4). Consequently, each tree age group developed a newly formed canopy containing comparable L1-L4 leaf layers. Therefore, the canopy layers reflected similar relative leaf developmental stages across tree age treatment, while the age of the underlying stem/root system differed. Unless otherwise stated, physiological and biochemical analyses were performed using 7-month-old trees. Trees grown in the phenotyping platform were used only for plant height measurements.

*Arabidopsis thaliana* wild-type (Col-0) and mutant (*stn7*, *tap38*, *npq1*, *npq4*, and *l17*) were grown under constant white light (130 μmol photon m^-2^ s^-1^, 8 h photoperiod, 23 °C /18 °C, 75% RH) for 5 weeks.

For outdoor measurements, 5 mature *Populus tremula* trees in natural stands on the Umeå University campus (N63°49’ E20°18’, Sweden) were measured weekly from late spring (28 May) when leaves had just emerged until leaf senescence and abscission in autumn (8 October). To reduce variation, for each tree, leaves were sampled from the outer protruding region of the lower canopy to minimize shading effects. Measurements were performed on randomly selected leaves from the same canopy region.

Independent trees of *Prunus maackii*, *Sorbus aucuparia*, and *Betula pendula* were followed from 11 August to 8 October (on Umeå University campus).

### Chlorophyll fluorescence measurements

State transition (ST): measured using Dual-PAM-100 (Walz, Germany) and ChloroSpec (the Netherlands,^44^). For Dual-PAM-100 measurements, qT was calculated from integrated chlorophyll fluorescence values, whereas for ChloroSpec measurements, qT was estimated from the area of the fluorescence emission spectra. Leaves were dark-adapted for 30 min, then exposed to weak red light ( ~ 10 μmol photon m^-2^ s^-1^, 15 min) to induce state 2. Subsequently, saturating pulses (8,000 μmol photon m^-2^ s^-1^, 300 ms) were applied every 5 min (three pulses total) while maintaining the same background illumination to measure F_m_ (S2). Leaves were then exposed to far-red light (15 min) to induce state 1, and F_m_ (S1) were also recorded (Supplementary Fig. 2). State transition capacity was calculated as previously described^45^:

1 $${{\rm{qT}}}=\frac{{{\rm{F}}}{{\rm{m}}}({{\rm{S}}}1)-{{\rm{F}}}{{\rm{m}}}({{\rm{S}}}2)}{{{\rm{F}}}{{\rm{m}}}({{\rm{S}}}1)\,}$$

Both instruments produced comparable results. For qT measurements in Arabidopsis rosette, a SpeedZen imaging system (Beam/API, France) was used with a comparable protocol.

To validate our protocol for ST measurement, we used *Arabidopsis* plants as a reference. As expected, *stn7* and *tap38* mutants showed negligible state transitions, while WT and other NPQ mutants showed normal levels, confirming that our measurements specifically captured ST but no other forms of quenching (Supplementary Fig. 2E).

Non-photochemical quenching (NPQ): recorded with Dual-PAM under increasing actinic light intensities (28-1723 µmol photon m^-2^ s^-1^, 10 min each). F_v_/F_m_ and PSII light use efficiency (Φ_PSII_, 150 μmol photon m^-2^ s^-1^) were determined as previously described^46^. Φ_PSII_ was calculated as:

2 $$\Phi {{\rm{PSII}}}=\frac{{{\rm{F}}}{{\rm{m}}}{{\mbox{'}}}-{{\rm{F}}}{{\rm{s}}}}{{{\rm{F}}}{{\rm{m}}}{{\mbox{'}}}\,}$$

Where F_m_’ is maximal fluorescence in the light-adapted state and F_S_ is steady-state fluorescence.

### Generation of *stn7* mutants

CRISPR-Cas9 vectors containing two guide RNAs targeting the *Populus* STN7 gene (Potrx. 065207g25331) were transformed into WT (T89) (Supplementary Fig. 3A,^47^). Genomic DNA from regenerated lines was amplified with primers (Supplementary Table 1) and was resolved on 1.5% agarose gels (Supplementary Fig. 3B). One line carried a 497 bp depletion in both alleles, while another had the same deletion in one allele and a second allele with an 1110 bp fragment that contained multiple small nucleotide deletions upon sequence alignment with the WT (T89) (Supplementary info.). Loss of STN7 function was verified by the absence of LHCII phosphorylation after PSII excitation and ST measurements using PAM (see Results).

### Thylakoid isolation

Thylakoids were isolated as described in refs. ^48,49^ with minor modifications. Miracloth (Merck Millipore, U.S.) was used to filter crushed leaves after homogenization. For state-specific isolation, greenhouse-grown leaves were dark-adapted for 30 min, detached, and hydrated by placing the petioles in water. Half of each leaf was exposed to far-red light for 30 min (state 1), and the other half was subsequently exposed to red light for 30 min (state 2). Thylakoids were isolated immediately, with NaF included in buffers to preserve phosphorylation status.

### Blue native PAGE and SDS second dimension

Blue native PAGE was performed using 3–12% BisTris native gels (Thermo Fisher Scientific, U.S.) Samples containing 30 μg chlorophylls were solubilized with 2% digitonin (Sigma-Aldrich, U.S.) at room temperature (21 °C, 10 min, gentle shaking) and centrifuged at 18,000 × g for 20 min. Aspen thylakoids have a different membrane composition than *Arabidopsis* requiring a higher digitonin concentration (2 vs. 1%). The pellet was discarded, and supernatants were loaded onto gels.

For the second dimension, BN gel strips were cut with a sharp blade and incubated in Laemmli buffer (62.5 mM Tris, 2% SDS, 10% glycerol, 2.5% 2-mercaptoethanol, 0.2% bromophenol blue) with gentle shaking for 1 h. Gel strips were then placed onto SDS-PAGE for the second dimension^50^. SDS gel (3.3% C, the weight percentage of cross-linker) was cast with 12% and 6% T (the total concentration of acrylamide and bisacrylamide) for resolving and stacking gel. The gel electrophoresis was run at 90 V (20 min) and 120 V (90 min).

Gels were stained with a QC Colloidal Coomassie solution (Bio-Rad).

### Western blot

Western blot was performed as previously described^31^. Sample was loaded onto the commercial purchased 12% pre-cast gel (Mini-Protein TGX 4561043, Bio-Rad). Primary antibodies (Agrisera, Sweden) included: anti-Lhcb1 (AS01004, diluted at 1: 2000), anti-Lhcb1-P (AS132704, diluted at 1: 8000), anti-Lhcb2 (AS01003, diluted at 1: 5000), anti-Lhcb2-P (AS132705, diluted at 1: 8,000), anti-PsaA (AS06172, diluted at 1:5000), anti-CP43 (AS111787, diluted at 1:6000), anti-D1(AS132668, diluted at 1:10,000), anti-CP26 (AS01009, diluted at 1:2000), anti-STN7 (AS164098), and anti-LHCA1(AS01005, diluted at 1:10,000).

### Pigment analysis

Leaf chlorophyll content was estimated with a CCM-300 meter (OPTI-SCIENCES, U.S.).

Chlorophyll concentration and compositions of thylakoid were determined by fitting absorbance spectra of 80% acetone extracts of pigment measured with a Shimadzu UV-2600i spectrophotometer (Japan) to individual pigment absorbance as previously described^51^. Extinction coefficients of the pigments were from^52^.

### Low-temperature fluorescence

Samples (OD < 0.05) were frozen in liquid nitrogen and measured at 77 K with a FluoroMax spectrofluorometer (HORIBA Scientific, U.S.). Excitation wavelength was 475 nm (2 nm slit), and the emission spectra were recorded from 600 nm to 800 nm at 1 nm resolution.

### Gas exchange measurement

Gas exchange was measured using an infrared gas analyzer (Li-6800, Li-Cor, Lincoln NE, USA). Net CO_2_ assimilation rate (*A*_n_) and effective PSII light use efficiency (Φ_PSII_) were measured under PPFD of 150 µmol m^−2^ s^−1^ (red actinic light and measuring light with a peak wavelength at 632 nm), air CO_2_ concentration of 400 µmol mol^−1^ and leaf temperature at 22 °C during the evaluations. The measurements were performed between 10:30 and 12:30 h.

### Transmission electron microscopy (TEM)

Leaf pieces were fixed in 4% paraformaldehyde and 2.5% glutaraldehyde in 0.1 M sodium cacodylate buffer (pH 7.4). State transitions were induced as in Thylakoid isolation. Sample preparation, embedding, sectioning, and staining were performed according to ref.^47^. Sections were imaged on a Talos 120 C TEM (FEI, Eindhoven, The Netherlands). ImageJ was used to analyze the membrane ultrastructure in the chloroplast. More than 26 chloroplasts and 300 grana of each sample (at least 3 independent leaves) were used for analysis.

### Shading experiment

To investigate whether reduced state transition capacity in lower leaves could be induced by shading alone, short aspen plants ( ~ 40 cm height) were placed beneath a dense canopy formed by taller aspens ( ~ 2.5 m height) grown in the greenhouse. Control plants were grown without canopy shading under the same greenhouse conditions. Shading treatment lasted for 12 days. qT measurements were subsequently performed on top and bottom leaves as described above for chlorophyll fluorescence measurements.

### Reporting summary

Further information on research design is available in the Nature Portfolio Reporting Summary linked to this article.

## Supplementary information

Supplementary Information

Reporting Summary

Transparent Peer Review file

## Source data

Source Data

## Data Availability

The data supporting the findings of this study are available on Figshare at DOI: 10.6084/m9.figshare.33070208. All data are publicly available without restriction. Source data are provided with this paper.
