# Supplementary material for: Leaf development regulates state transition capacity in trees
Source: Nat Commun. 2026 Aug 6;17:7926. doi: 10.1038/s41467-026-76469-5 (PMC13447977; doi:10.1038/s41467-026-76469-5)
Supplement: Supplementary file 1 [file 41467_2026_76469_MOESM1_ESM.pdf]

Supplementary Figures

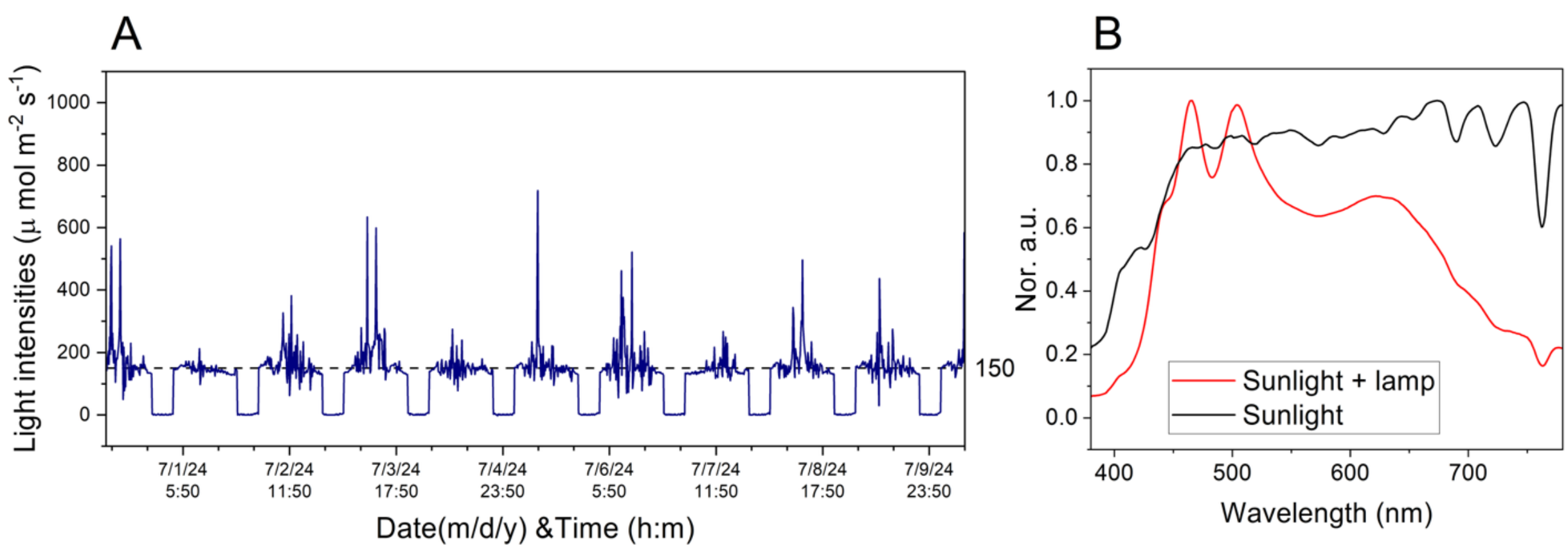

Fig. S1. Light environment in greenhouse. (A) Representative fluctuating light intensities in the *aspen* growth greenhouse recorded over a 10-day period. (B) Representative emission spectra of light in the greenhouse, with and without additional lamp light.

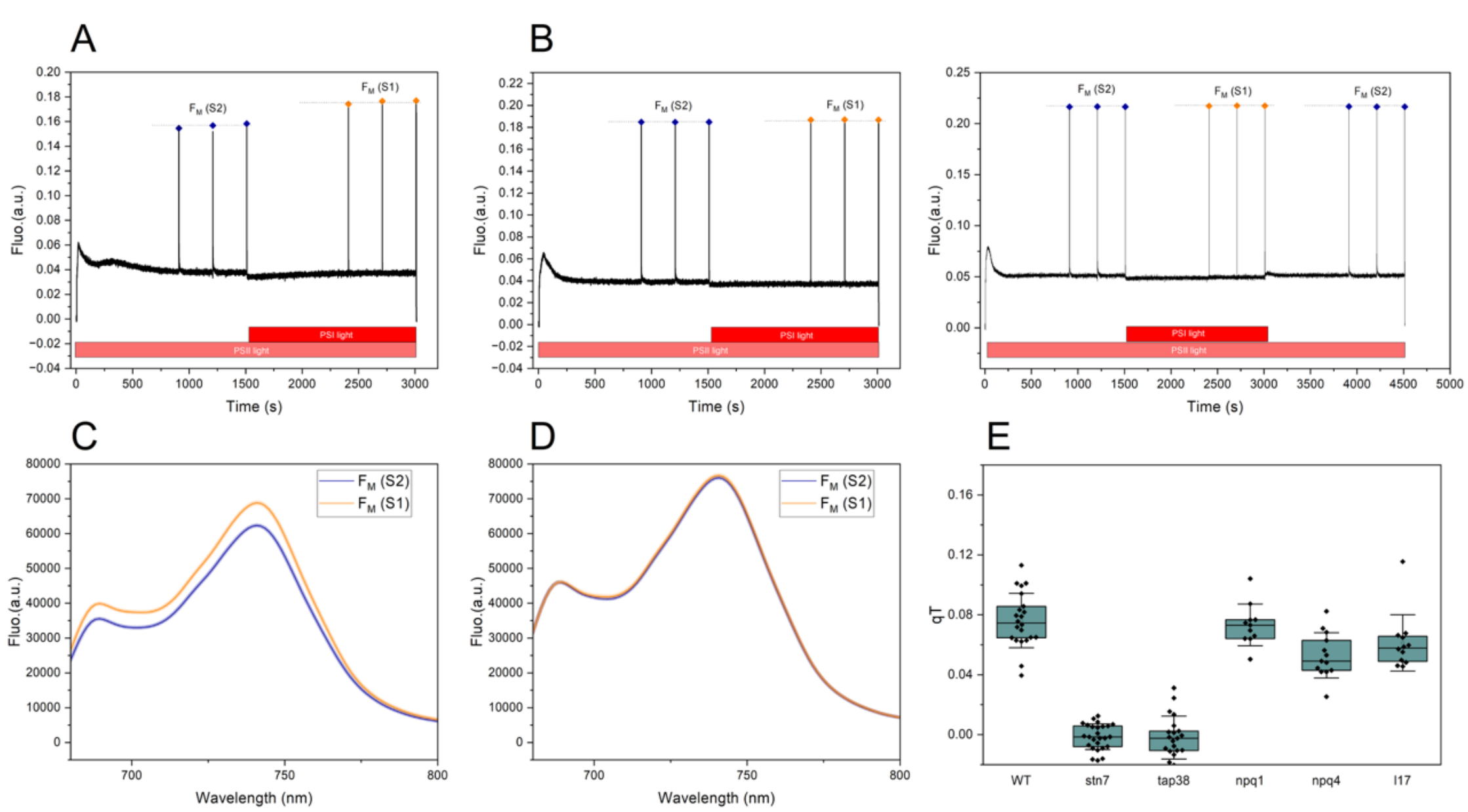

Fig. S2. Validation of state transition (ST) measurements in aspen and *Arabidopsis*. (A) Dual-PAM measurement of ST in an aspen T89 L1 leaf, showing fluorescence traces during state 2 to state 1 induction. (B) ST measurement with Dual-PAM on an L4 leaf under two-phase (left) and three-phase (right) actinic light protocols. (C) Fluorescence yield and spectra of state 1 (ST1) and state 2 (ST2) on an L1 leaf measured with ChloroSpec (n = 3, independent biological replicates). (D) Fluorescence yield and spectra of ST1 and ST2 on an L4 leaf measured with ChloroSpec (n = 3, independent biological replicates). (E) ST capacity in different *Arabidopsis thaliana* genotypes (n = 20 WT, n = 25 stn7, n = 19 TAP38, n = 10 NPQ1, n= 11 npq 4, n =10 L17). Individual data points are shown for each independent biological replicate. Data were presented as box plots, with the median indicated by the dashed lines, the lower and upper bounds of the box representing the 25th and 75th percentiles, respectively, and the whiskers representing the standard deviation.

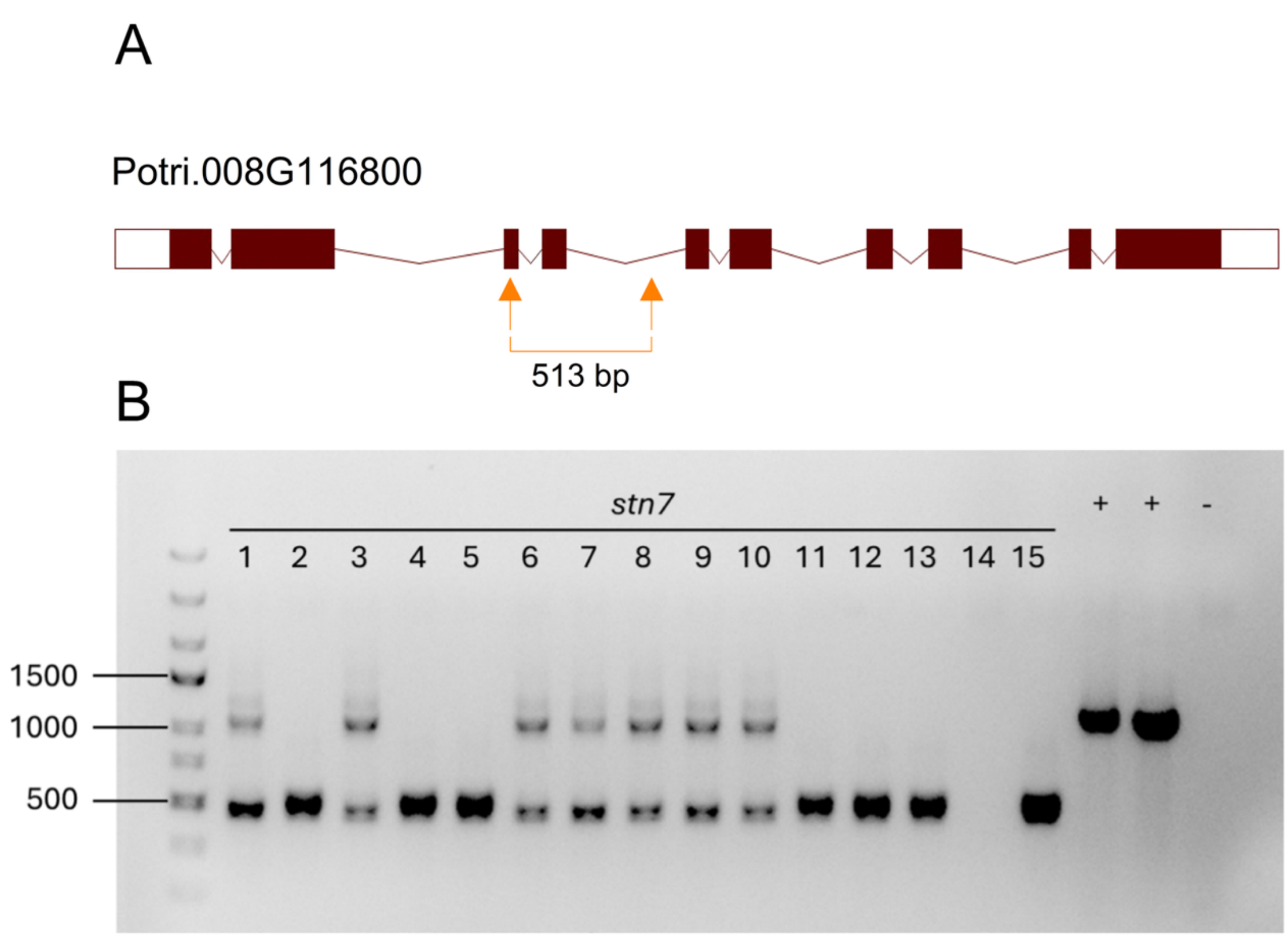

Fig. S3. Generation and molecular validation of aspen *stn7* mutants. (A) Schematic representation of the STN7 locus showing the position of sequence deletion. Targeted regions correspond to sgRNA sites. (B) Gel electrophoresis after PCR with genotyping primers (c.f. Tab.S1) for 15 biological replicates of two independent *stn7* lines. The STN7 gene is detected at 1110 bp in the wild-type background and with a deletion at 497 bp in the *stn7* lines indicating that it is a deletion mutant line. Here we have a mix of heterozygous and homozygous lines for the *stn7* gene.

"+" indicates wild-type T89 template and "-" indicates a control with no added template for the PCR reaction. Lanes 2, 4, 5, 11, 12, 13, and 15 are individual plants from *stn7* line 1. Lanes 1, 3, 6, 6, 8, 9, 10 are individual plants from *stn7* line 2.

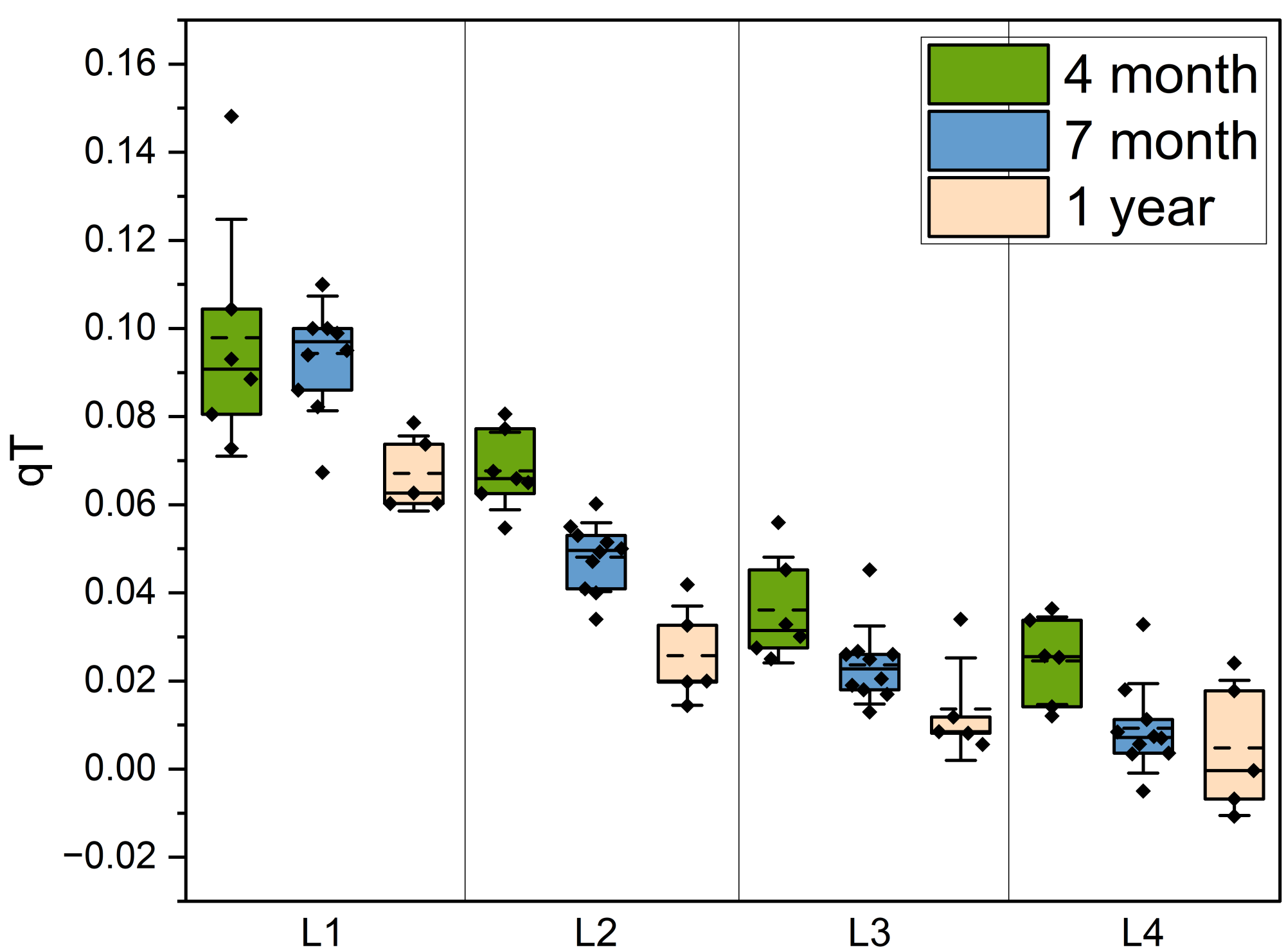

Fig. S4. Vertical distribution of state transitions across different canopy layers of aspen T89 leaves collected from comparable height of trees but in different ages (n = 5, independent biological replicates). Data were presented as box plots, with the median indicated by the dashed lines, the lower and upper bounds of the box representing the 25th and 75th percentiles, respectively, and the whiskers representing the standard deviation.

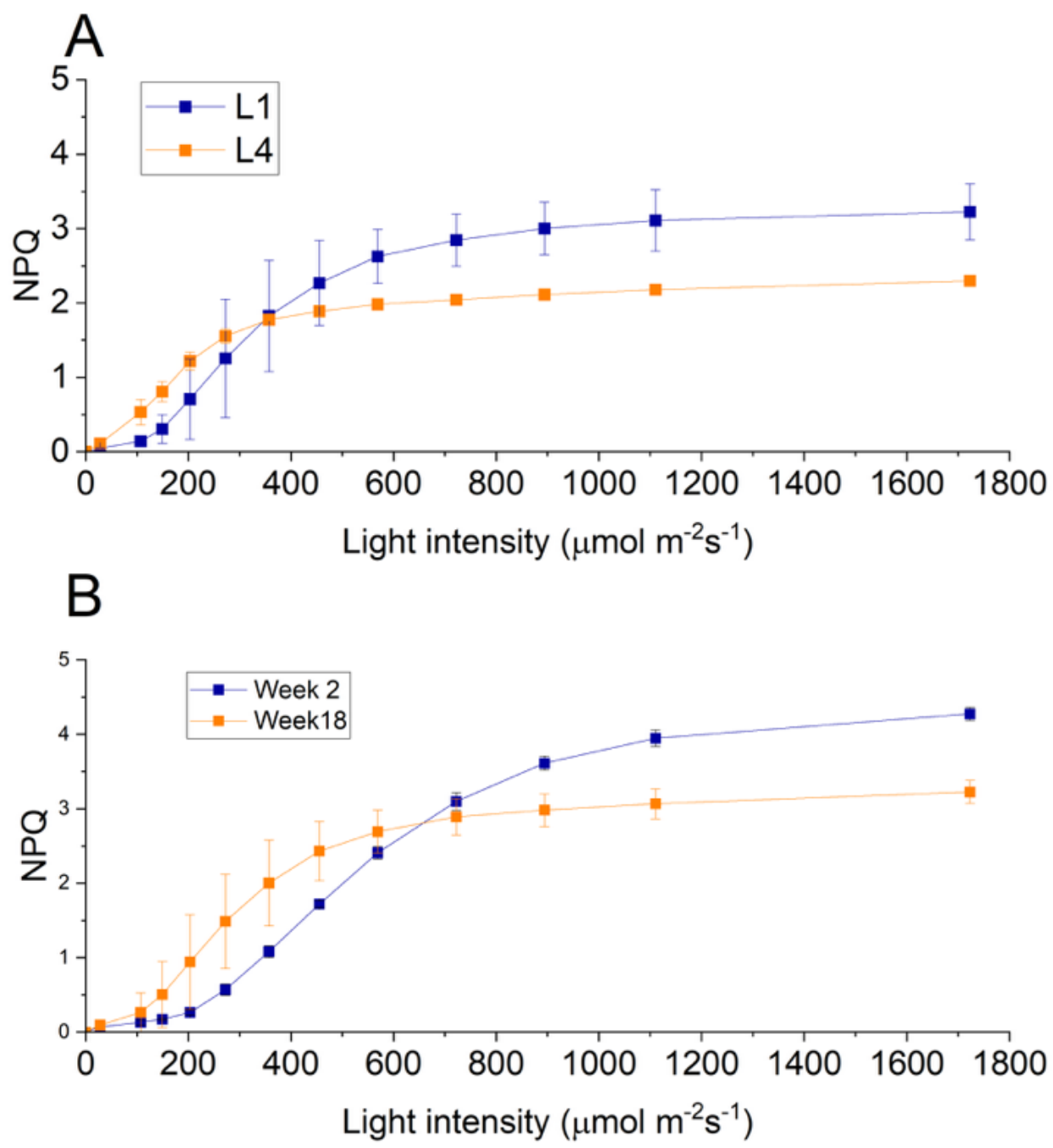

Fig. S5. Vertical distribution of non-photochemical quenching (NPQ) across different canopy layers of indoor aspen (T89) leaves and different seasons of aspens grown in the field. (A) NPQ light response curve of L1 and L4 of indoor aspens (T89). (B) NPQ light response curve of outdoor aspen leaves at different weeks (n=3, independent biological replicates).

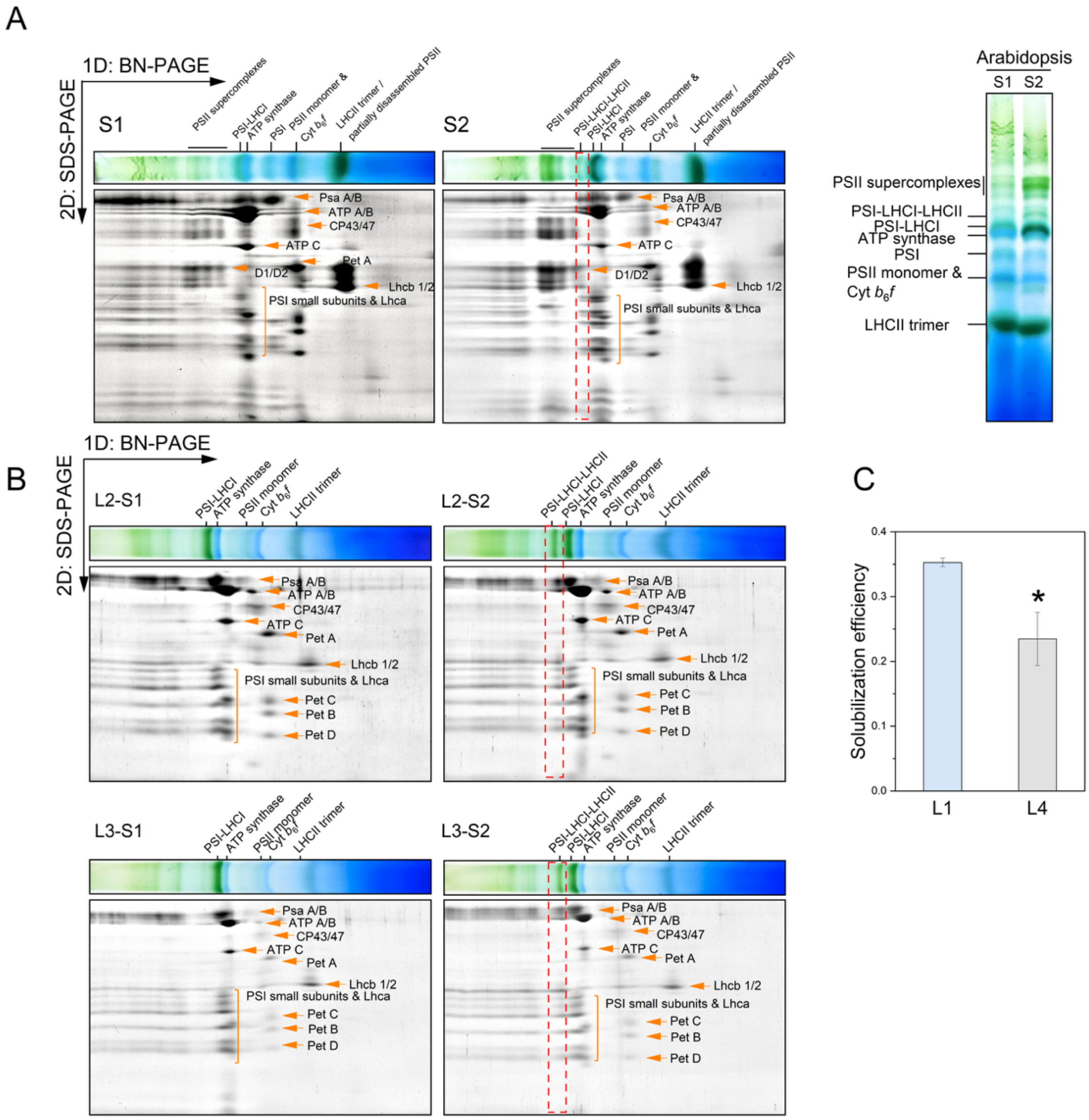

Fig. S6. Two-dimensional BN/SDS-PAGE analysis of thylakoids isolated from *Arabidopsis* and aspen under different states. (A) BN/SDS-PAGE analysis of thylakoids isolated from *Arabidopsis*. (B) Two-dimensional SDS-PAGE analysis of thylakoid membranes isolated from L2 (top panel) and L3 T89 (bottom panel) aspen leaves. State 1 on the left and state 2 on the right. (C) Quantification of digitonin solubilization efficiency in L1 and L4 thylakoids estimated from chlorophyll content remaining in the insoluble pellet fraction following centrifugation (n=3, one way ANOVA, $P = 0.00791$). Full uncropped gel images are provided in Source Data file.

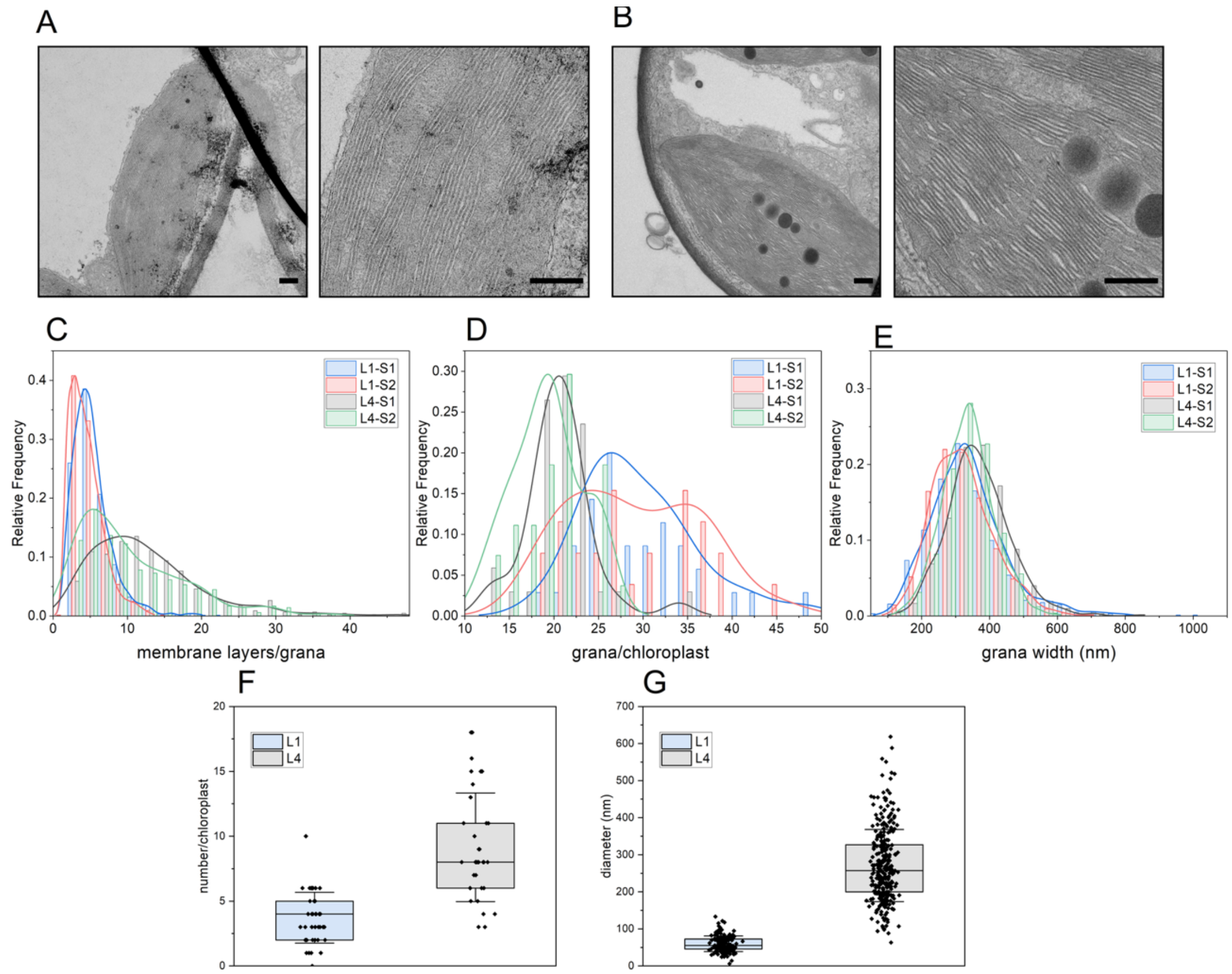

Fig. S7. Transmission electron microscopy (TEM) analysis of chloroplasts in the top (L1) and bottom (L4) layers of aspen T89 leaves. (A-B) Representative TEM images of chloroplasts from L1 (A) and L4 (B) in state 2. Scale bar = 300 nm. (C) Distribution of thylakoid membrane layers per grana. (D) Grana number per chloroplast. (E) Grana width (nm). Curves represent kernel density estimates of the distributions. Quantification of plastoglobuli (F) abundance per chloroplast (n =42 in L1, n = 34 in L4, independent biological replicates) and (G) diameter in chloroplasts in L1 and L4 leaves (n = 156 in L1, n = 311 in L4, independent biological replicates). Data were presented as box plots, with the median indicated by the dashed lines, the lower and upper bounds of the box representing the 25th and 75th percentiles, respectively, and the whiskers representing the standard deviation.

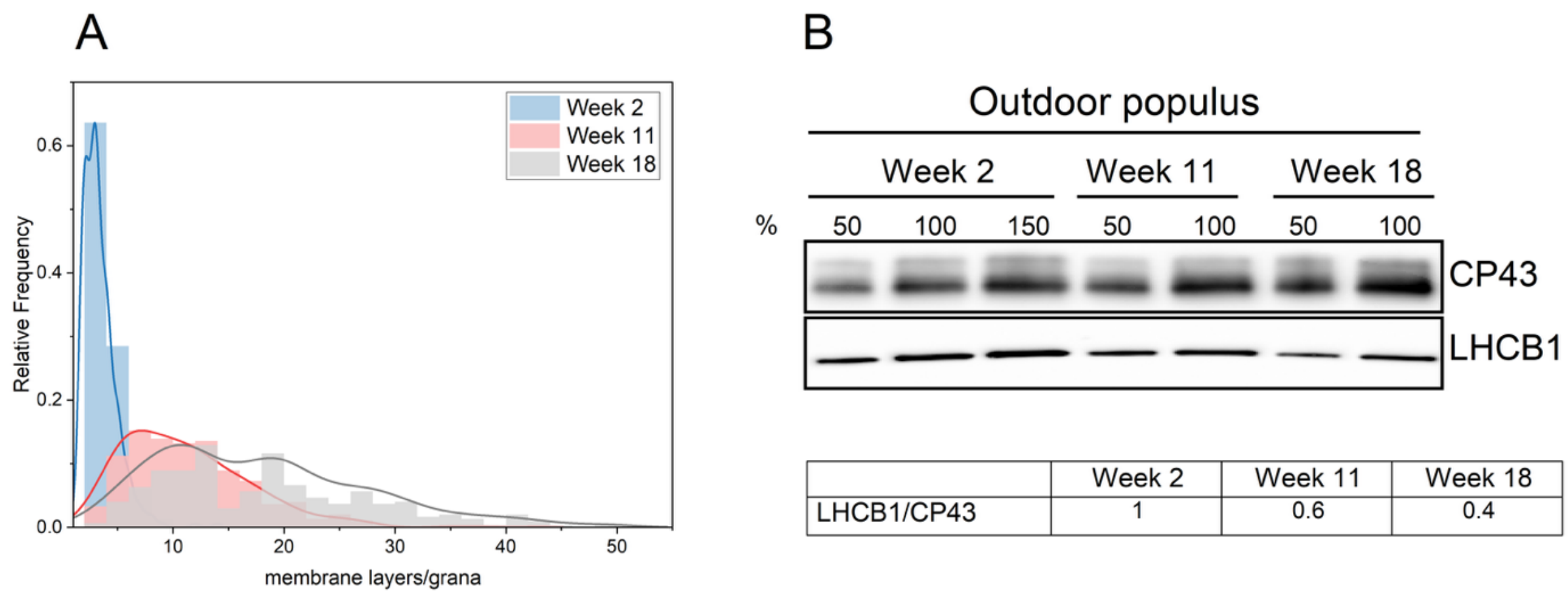

Fig. S8. Seasonal changes in grana architecture and LHCII abundance in field-grown aspens. (A) Quantification of thylakoid membrane layers per granum from transmission electron micrographs (n = 302 in week 2 and week 11, n = 301 in week 18, independent biological replicates). Each sample contains (n>300) grana. (B) Western blot analysis of Lhcb1 and CP43 used to estimate relative LHCII abundance across the seasonal time course.

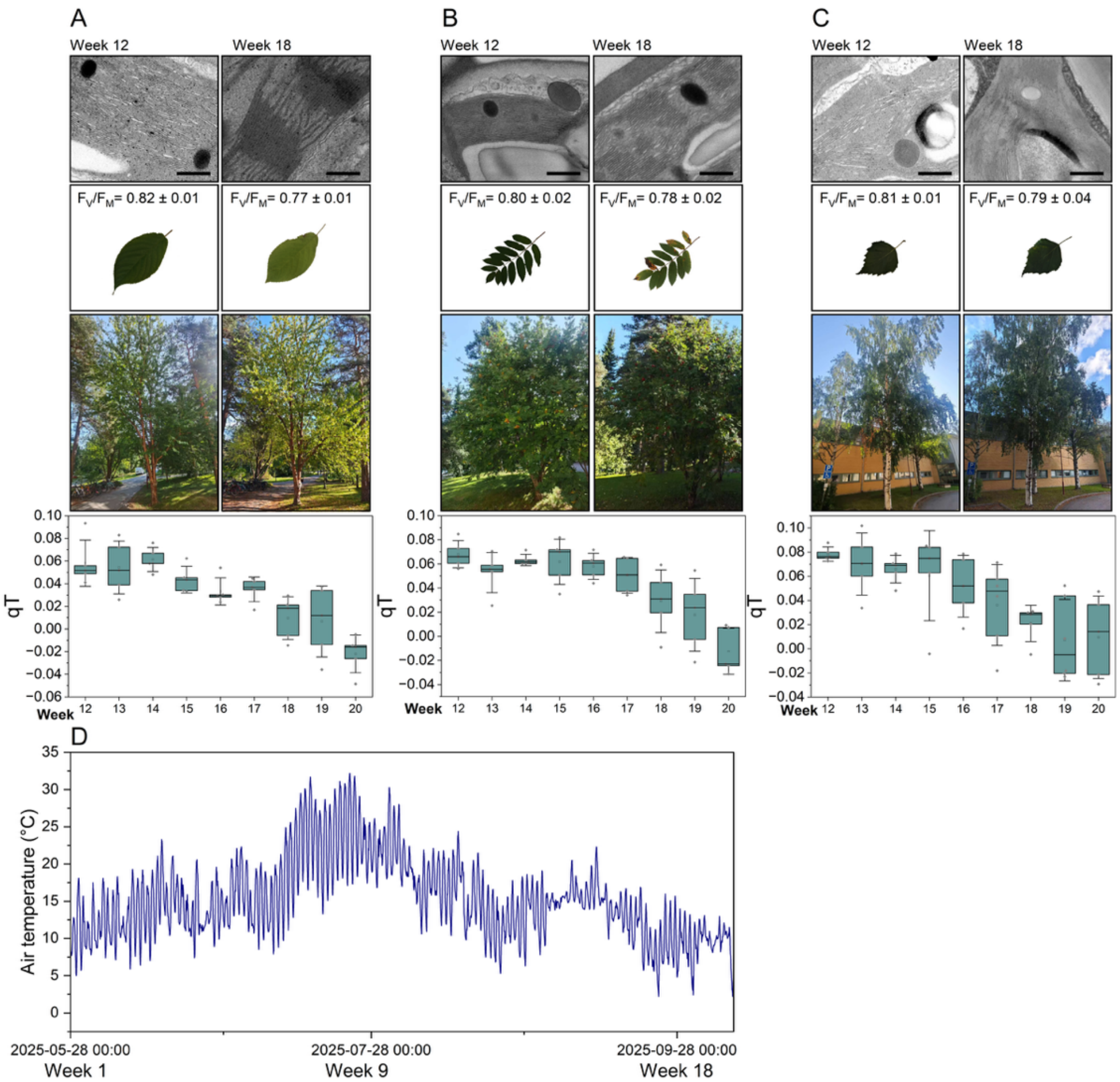

Fig. S9. Seasonal dynamics of state transitions in mature outdoor trees. (A) *Prunus maackii*. (B) *Sorbus aucuparia*. (C) *Betula pendula***.** Center panel: representative photographs of the relevant tree in Umeå, Sweden, taken from summer (12th Aug) to late autumn (19th Sep) 2025, alongside representative images of the leaves collected at different time points. Top panel: representative transmission electron micrographs of chloroplasts sampled in early August and late September; scale bars = 1 µm. Bottom panel: seasonal changes in state transition capacity (n=5, biologically independent samples) plotted over time. Data were presented as box plots, with the median indicated by the dashed lines, the lower and upper bounds of the box representing the 25th and 75th percentiles, respectively, and the whiskers representing the standard deviation. (D) Seasonal ambient temperature in Umeå, Sweden. Temperature data were obtained from the Open-Meto historical weather database.

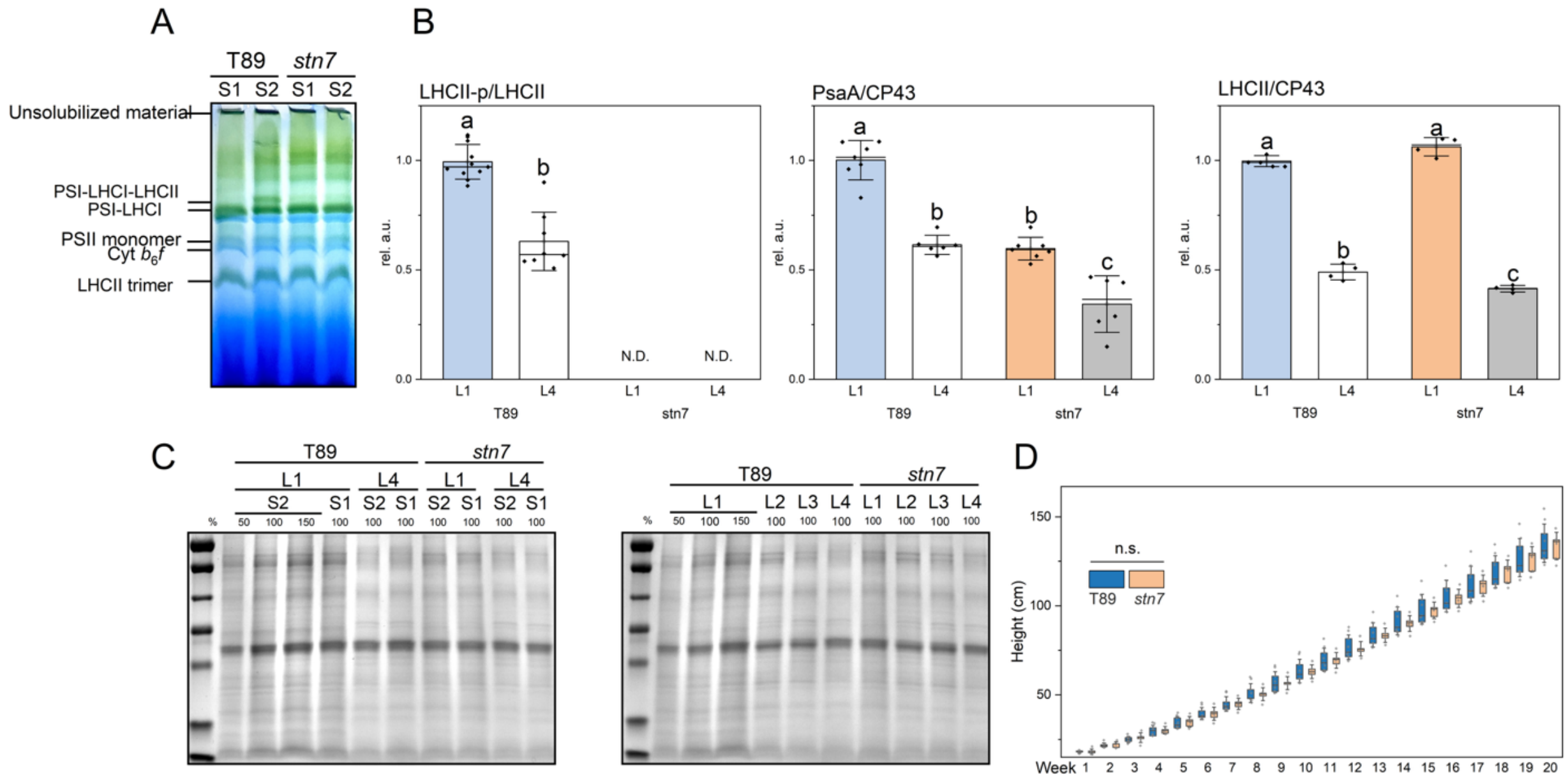

Fig. S10. Biochemical and phenotypic characterization of aspen *stn7* mutants. (A) BN-PAGE of aspen thylakoids isolated under state 1 and state 2. (B) Quantitative western blot analysis of thylakoid protein components in L1 and L4 of T89 and *stn7* mutant (normalized to L1 from T89 in state 2). Total LHCII was estimated from the combined immuno signals of Lhcb1 and Lhcb2, while phosphorylated LHCII was estimated from Lhcb1-p and Lhcb2 immuno signals. Data were presented as bar plots, with the median indicated by the solid lines, the whiskers representing the standard deviation. (C) Coomassie-stained SDS-PAGE gel as loading control. (D) Plant height of T89 and *stn7* grown under constant light (in phenotyping platform). Comparison of plant height between T89 and *stn7* mutant trees grown under constant light conditions (n = 9 in T89 and n = 6 in *stn7*, independent biological replicates, one-way ANOVA). Data were presented as box plots, with the median indicated by the dashed lines, the lower and upper bounds of the box representing the 25th and 75th percentiles, respectively, and the whiskers representing the standard deviation.

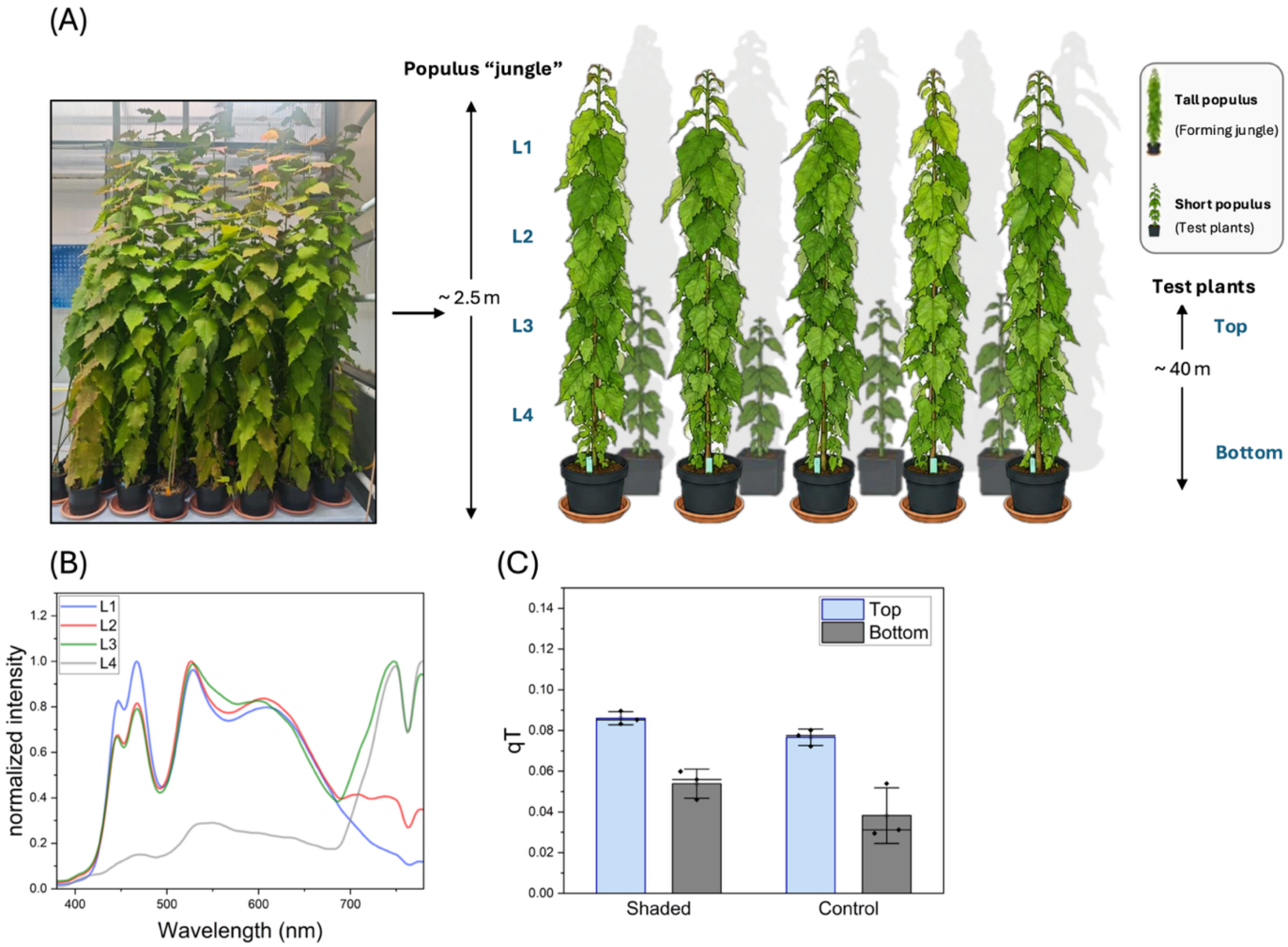

Fig. S11. Shading experiment on developing aspen trees. (A) Schematic of the shading experiment. Tall aspens (~2.5 m) were used to generate a dense "jungle", while shorter aspens (~40 cm) were placed beneath the canopy to experience shading conditions (B) Normalized light spectra measured from leaves at different canopy positions (L1 to L4) with the "jungle". (C) qT measured in top and bottom leaves of the shorter test plants grown either under shaded conditions ("Shaded") or without canopy shading ("Control") for 12 days (n=3). Data were presented as bar plots, with the median indicated by the solid lines, the whiskers representing the standard deviation.

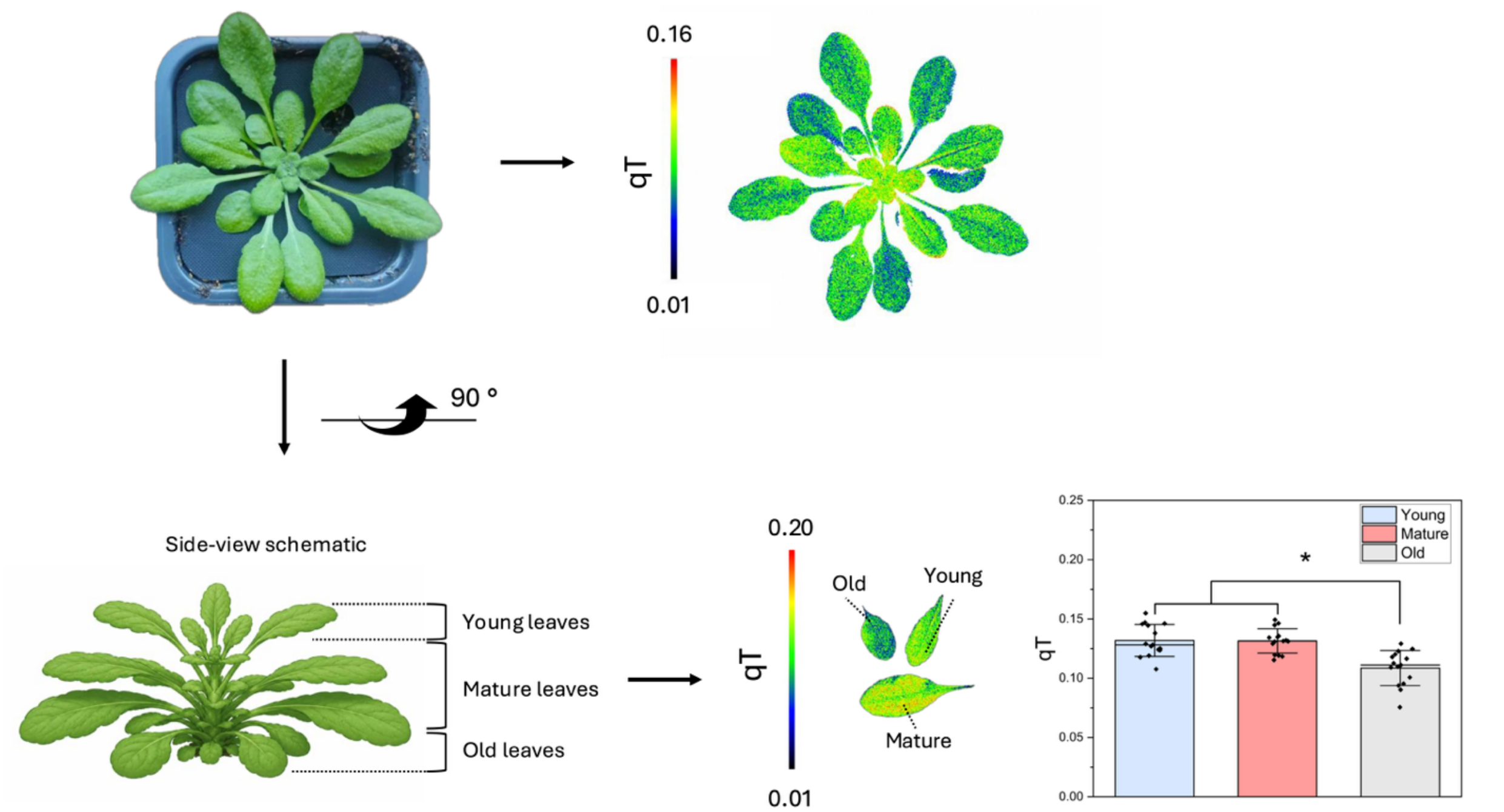

Fig. S12. Developmental analysis of qT across *Arabidopsis* (Col-0) rosette leaves using fluorescence imaging. Top: representative qT imaging of an intact arabidopsis rosette measured using the SpeedZen fluorescence imaging system. Bottom: schematic illustration of the leaf layers. Rosette leaves were separated into three developmental groups (young, mature, and old leaves), arranged on the same flat platform to minimize effects of leaf angle and self-shading, and analyzed for qT. Representative qT images and quantification are shown on the right (n = 15, one-way ANOVA, F (2, 42), $P < 0.0001$). Data were presented as bar plots, with the median indicated by the solid lines, the whiskers representing the standard deviation.

**Mutant**	**sgRNAs sequence**	**Targeted gene**	**Targeted gene ID (T89)**	**Genotyping primers**	
stn7	GTAGAGGTCTAAACTCGAAG	State Transition 7(STN 7)	Potrx065207g25331	Forward	GTGTCTACGTGTCATCTGTGG
	GTTGGGGAAGAAGTTGGGCG	Ser/Thr protein kinase		Reverse	ATCCGGTCATCCCATGAAAC

Tab. S1. sgRNA & genotyping primer sequences used for generating and testing CRISPR mutant lines.

Leaf layers	PSI-LHCI-LHCII/(PSI-LHCI + PSI-LHCI-LHCII)
L1	0.44
L4	0.21

Tab. S2. Densitometric quantification of PSI-LHCI-LHCII complex abundance in representative BN-PAGE image (Fig.2) from L1 and L4 thylakoid. Relative PSI-LHCI-LHCII abundance was calculated as PSI-LHCI-LHCII/(PSI-LHCI + PSI-LHCI-LHCII) based on ImageJ densitometric analysis.
