# Supplementary material for: Leaf development regulates state transition capacity in trees
Source: Nat Commun. 2026 Aug 6;17:7926. doi: 10.1038/s41467-026-76469-5 (PMC13447977; doi:10.1038/s41467-026-76469-5)
Supplement: Supplementary file 3 [file 41467_2026_76469_MOESM3_ESM.pdf]

nature portfolio

Peer Review File

Leaf development regulates state transition capacity in trees

Corresponding Author: Dr Chen Hu

This file contains all reviewer reports in order by version, followed by all author rebuttals in order by version.

Version 0:

Reviewer comments:

Reviewer #1

(Remarks to the Author)
"Leaf development regulates 1 state transition capacity in trees" is an interesting study that investigates state transitions in aspen leaves of different age, and compares a wild type hybrid aspen with its Stn7 KO that lacks state transitions. Three aspects make it specifically interesting: 1. Measurements on trees instead of Arabidopsis thaliana. 2. Aspect of leaf age is addressed. 3. Stn7 mutant is generated in aspen, second species after Arabidopsis. As trees are far more important for carbon fixation than Arabidopsis, this is a relevant topic to study. The work is original and combines greenhouse measurements with outdoor measurements, which make the relevance larger.

The manuscript could strongly benefit from more clearly explaining what has been done. In the current form the reader lacks some information to judge and interpret the data. Below I address, in the order of the manuscript, the points that I think need improvement. Minor issues are mixed with larger points.

Line 8 "photosystem II and I" you seem to follow the order of LET, but for the balance this does not matter, I suggest to write photosystem I and II.
Line 18 "reduced light-use efficiency in young leaves," you measure the PSII operating efficiency, while some readers might interpreted this as light-use efficiency a more common assumption might be CO2 assimilation per photon.
Line 18-19 "slower growth under fluctuating but not constant light. These findings indicate that ST is important for performance under dynamic light conditions" The light intensity fluctuations are not the only difference between the greenhouse and the climate room, the light spectrum and changes therein are also different.
Line 37 "on electron transport chain" -> of the electron transport chain
Line 99-100: "Although L1 leaves obviously had not yet a fully developed photosynthetic apparatus they were photosynthetically very active" why is this obvious? It looks like they have all photosynthetic components required? If you consider that L1 leaves have a lower Chl content, than the assimilation per absorbed photon might even be higher than for L2 leaves.
Line 107 "Comparisons between trees where shoots were 4 months, 7 months, and 1 year, grown" please explain for those who do not work with aspen what age the leaves have in this case. The L1 leaves are the newly formed leaves, which are always younger than the L4 leaves. But how old are L1 leaves in a 1 year grown aspen? And L2 leaves?
Fig. 1 is this data from 4 months old trees?
Throughout the manuscript T89 vs Stn7 is used, but Stn7 is generated from the T89. I think it would be better to say WT (T89) vs Stn7.
Fig. 1E in the M&M I can find the assimilation is measured at 150 uE, but I cannot find the light spectrum that was used, e.g. red/blue ratio preferably with wavelengths. Please add.
Fig 4. Would it be possible to provide temperature data in the SI? I don't know if this is recorded in a weather station? If the temperature in October was low, than this could also be a reason for reduced qT. This might be added to the discussion.
For the outdoor aspen measurements, how were the leaves assessed? What location in the canopy? Was any spectral data recorded? Is it likely that the leaves became shade adapted? The levels of Stn7 decrease in A.thaliana under long-term state 1 conditions (Willig, 2012 www.plantphysiol.org/cgi/doi/10.1104/pp.111.187328). Maybe something similar happens in aspen?
Line 195-196 "Also, 196 the BN-PAGE results (Fig. 2A) were not significantly different." In the example shown there is far less PSI-LHCII band relative to the PSI band in L4. What is the definition of "significant" used here?
Fig. 5C what is the colour/spectrum of light used to measure Phi(PSII)?
For 5E it is mentioned after 4 months growth in greenhouse, are the other measurements made on the same plants?
Measuring tree height is non-invasive, but is there also dry-weight or fresh weight data collected? The shape of the canopy

could be changed.
Line 226 use abbreviation for PSI and PSII.
Line 250 “for example, if there is a bottleneck at the PSII side rather than” please explain what you mean, e.g. if PSI is over-excited rather than PSII?
Line 316 “photoperiod Plants” -> photoperiod. Plants
Line 326: how was the canopy region defined?
Line 330: was qT only quantified from the Dual-PAM-100 measurements, or also from the ChloroSpec?
Line 332-333: It reads like the light conditions lasted for 15 minutes, but it is 25 minutes, please indicated that the saturating flashes occur after the 15 minutes of continuous illumination. Please provide information on the durations and intensity of the saturating flash.
Line 335: Vaygan is not the primary source for the equation and please provide the equation.
Line 3412: please provide the formula for Phi(PSII) that was used and mention the light intensity also here.
396 TEM methods. Duration of fixation should be mentioned, as well as further protocol to embed and stain the sample. Or refer to a published protocol. It should also be mentioned how many different leaves were investigated, one can easily take 26 chloroplast from one leave, but that is only 1 biological replication.

SI
Fig. S2 “showing fluorescence traces during state 1 to state 2 induction.” First state 2 is induced and next state 1.
Fig. 2B right the last two saturating pulses appear very high, how can this be?

Fig. S8 what is the bin-size in the histogram? I count 7-8 bind per 10 membrane layers/grana, indicating that one bin is ~1.3 membrane. On the other hand the 3 data points are shown one after the other indicating bins of 3-4? Please make clear what the bins are. You can e.g. use transparent filling, to show the data on the same bins, or have the three bars together at the bin and have white space in between.

Fig. S10B please show an example of the blots. It is not clear how LHCII-p/LHCII was obtained, as LHCII is composed of Lhcb1-3. Was total LHCII estimated from the SDS-PAGE?

The PsaA/CP43, the Chl a/b ratio and the thylakoid organisation indicate strong adaptation to shade light in L4. This is could be discussed more in the Discussion. It could be that adaptation to shade light provides a more precise explanation of the results than leaf development in general.

Reviewer #2

(Remarks to the Author)
State transition (ST) is an important mechanism of adaptation to changing light conditions in photosynthetic organisms which has been mostly studied in Arabidopsis and in the unicellular alga Chlamydomonas. In this study the authors have examined ST in the tree model species aspen. They have quantified ST across vertical leaf layers of aspen subjected to long-term fluctuating light. Moreover they have also generated by CRISPR/Cas9 mutants deficient in ST to functionally test the role of ST in trees. Finally the authors have examined seasonal changes of ST in mature aspens and three other tree species grown under natural field conditions. The major conclusions of this study, based on physiological, biochemical, and ultrastructural analysis, is that in trees the leaf developmental stage impacts ST more than changing light conditions although it is not clear how this developmental control operates. This study also shows that in trees, the loss of the STN7 protein kinase has similar effects as in Arabidopsis, but surprisingly that in older leaves there is still appreciable LHCII phosphorylation with little ST. Overall, these are valuable results which extend our knowledge on the dynamic photoregulation of thylakoid architecture in angiosperms beyond Arabidopsis.

Although the technical quality of this study is high and the results are interesting, in my opinion it does not provide sufficient novel insights into the regulatory mechanisms of ST to justify publication in Nature Communications. I suggest that the authors submit their manuscript to a more specialized plant journal.

Reviewer #3

(Remarks to the Author)
The manuscript investigates state transitions across leaf developmental stages in hybrid aspen and other tree species. The study addresses an interesting biological question and presents a valuable dataset; however, several aspects of the work would benefit from strengthening before the manuscript is ready for publication, including additional validation experiments that appear feasible within the existing framework and a more thorough engagement with the current mechanistic literature on state transitions.

Major Comments
Line 14
"Reduced ST correlated with increased grana stacking, but not with the LHCII phosphorylation, suggesting developmental remodeling may physically constrain LHCII redistribution."

This claim appears overly speculative given the data presented. The authors use qT as their primary measure of state transitions (ST) - qT is a parameter based on the changes of PSII fluorescence intensity in PSI- and PSII-specific light, and is related to the changes in PSII antenna cross section. It is important to note that an increase in qT does not necessarily imply long-distance relocation of LHCII from grana to stroma thylakoids; simple detachment of LHCII from the PSII core is sufficient to produce an increase in qT. The statement should therefore be revised to reflect what the data can and cannot support, and speculative language about the physical constraint of LHCII redistribution should either be substantiated or toned down accordingly.

Line 47
"Under PSII-light, the plastoquinone pool is reduced and the major light complexes II (LHCII) are phosphorylated by the chloroplastic serine/threonine-protein kinase STN7, and subsequently relocate from PSII to PSI, increasing the PSI antenna size (Cutolo et al., 2023; Jarvi et al., 2011; Longoni et al., 2019; Schiphorst et al., 2022; Wunder et al., 2013). This state is called state 2 (Bellafiore et al., 2005)."
This description of the state transitions mechanism is somewhat outdated, as it does not account for more recent evidence demonstrating only partial migration of LHCII between grana and stroma thylakoid compartments. Thorough analyses have indicated that long distance LHCII migration is considerably more limited than the classical model suggests. The authors should consider incorporating and discussing the revised perspective on this process, including, in particular, the work from Matt Johnson's laboratory.
Additionally, there is an internal terminological inconsistency that warrants attention. The definition given here for state 2 implies full LHCII relocation, yet throughout the manuscript, state 2 is operationally identified via qT measurements, which reflect a decrease in PSII antenna cross-section (i.e., LHCII detachment from PSII core) rather than confirming physical relocation. Similarly, the 77 K fluorescence emission data - cited as evidence for state transitions - reports on increased LHCII association with the PSI-LHCI complex, which is mechanistically distinct from qT. The authors are encouraged to be more precise and consistent in their use of terminology across the manuscript, clearly distinguishing between LHCII detachment and LHCII relocation where relevant.
Line 73
"We demonstrate that state transition in trees is strongly dependent on the leaf developmental stage, which has a stronger effect than light environment alone."
The authors make a strong claim about the primacy of developmental stage over light environment, yet the experimental design does not include complementary experiments in Arabidopsis that would help contextualize this finding within the broader literature. Including data from Arabidopsis rosettes - where leaf position serves as a well-established proxy for leaf age - would provide a useful bridge between the extensive existing literature on Arabidopsis state transitions and the novel tree-focused work presented here. Specifically, PAM imaging-based qT measurements across leaves at different rosette positions could help disentangle whether the observed developmental decline in state transition capacity is a tree-specific phenomenon or a more general feature of angiosperm leaf maturation. This would considerably strengthen the conclusions of the paper.
Furthermore, the authors do not appear to have measured the light spectra actually reaching leaves at different canopy layers. Light filtered through upper leaves is spectrally enriched in far-red wavelengths, which could independently influence state transition induction. The authors should address whether the observed canopy gradient in state transitions might be partly attributable to differences in spectral composition rather than (or in addition to) leaf developmental stage.

Line 123
"Under state 2, L1 thylakoids displayed PSI-LHCI-LHCII complexes, which were absent in state 1 (Fig. 2A). This complex was also present, although at a lower level, in L4 in state 2 (Fig. 2A)."
Before drawing conclusions from the BN-PAGE data, the authors should verify and report the efficiency of digitonin solubilization across the different variants. Specifically, was chlorophyll content measured in the pellet fraction following centrifugation after digitonin treatment? A comparison of this efficiency across leaf positions and state conditions is essential for valid interpretation. Additionally, a quantitative analysis of the BN-PAGE data - such as calculating the intensity of the PSI-LHCI-LHCII band relative to the PSI-LHCI band - would considerably strengthen the claim, as the overall lane intensities across the studied variants appear to differ, which complicates direct comparison of band patterns.

Line 128
"Although the relative abundances of some individual proteins varied (Fig. 2C)."
No quantitative analysis is provided in the manuscript to support this statement. Additionally, please clarify how the samples loaded in each lane were equalized (e.g., on a chlorophyll, protein, or other basis).

Line 147
"Additionally, plastoglobuli were observed in L4 leaves (Fig. 3B, Fig. S7). Plastoglobuli are associated with older aspen leaves and accumulate as the leaves enter senescence; however, they were less abundant in L4 than in fully senescing aspen leaves (Keskitalo et al., 2005)."
This statement and the subsequent description give the misleading impression that plastoglobules are exclusive to L4 leaves. However, as is clearly visible in the TEM images presented in the manuscript, plastoglobules are also present in younger leaves - albeit at smaller sizes. The developmental increase in plastoglobule size is a well-documented phenomenon in chloroplast biology, and plastoglobules are in fact a constitutive feature of plastids at all developmental stages, including etioplasts. The text should be revised to reflect this accurately. Furthermore, the authors should provide quantitative data on plastoglobule size (diameter) and abundance per chloroplast across the different leaf positions to support their comparative statements.

Fig. 3

The data presented here illustrate the well-known developmental progression of thylakoid network organization - an initial increase in grana diameter followed by a build-up of membrane layers - a pattern that has previously been described in crop plants and other species. However, no references are cited to acknowledge this prior literature. The authors should include appropriate citations documenting the developmental trajectory of thylakoid architecture to contextualize their findings.

Fig. 4 — Bottom panel: seasonal changes in state transition capacity
To ensure that the seasonal changes in qT reflect genuine variation in state transition capacity rather than shifts in the stoichiometry of the photosynthetic machinery, the authors should provide supporting data demonstrating that the ratio of PSII core complexes to LHCII antenna is stable across the sampling time points. Changes in the PSII/LHCII ratio could significantly influence qT values independently of any modulation of state transitions per se, and this potential confound should be addressed.

Line 244
"Our data indicate that the limitation of ST in older/lower leaves is not simply due to the absence of LHCII phosphorylation or PSI association (with LHCII). In L4 leaves, we still observed substantial LHCII phosphorylation (60% of T89) and PSI-LHCI-LHCII assemblies under state 2 conditions, yet chlorophyll fluorescence measurements showed only little ST."
While this is an intriguing observation, the authors do not offer a mechanistic hypothesis to explain the apparent disconnect between LHCII phosphorylation, PSI-LHCI-LHCII assembly, and the qT-based assessment of state transitions. A more thorough discussion of possible explanations would be valuable. Furthermore, as noted above, this apparent paradox may also partly reflect the definitional ambiguity between qT (which reports on LHCII detachment from PSII) and state transitions as defined by LHCII association with PSI. A more careful treatment of these distinctions in the discussion would help the reader interpret the data appropriately.

Line 289
"As it has been suggested that the STN7 kinase may have other roles (Poudyal et al., 2020) in addition to regulating state transitions, the question is if the lack of ST or some other STN7 functions is causing this."
This sentence is unclear and would benefit from revision.
Line 299
"This decline occurs despite sustained LHCII phosphorylation, suggesting that the functional extent of state transitions is influenced by the structural maturation of the thylakoids."
This statement is problematic for reasons discussed above: if state transitions are operationally defined throughout this manuscript by qT measurements - which reflect changes in PSII antenna cross-section rather than direct structural rearrangements - then invoking grana metrics as an explanatory variable requires careful justification. The authors should either provide a mechanistic link between grana structure and qT or qualify their interpretation accordingly.

Minor Comments
Abstract
"reports on the regulation and ecological significance of ST in other angiosperms remain largely unknown"
This sentence implies that the present work will comprehensively address the ecological significance of state transitions, which it does not. The abstract should be revised to more accurately reflect the scope of the study.

Terminology: FV/FM
"maximum PSII quantum yield (FV/FM)"
This notation is outdated. The authors should use the updated nomenclature as recommended in the recent guidelines (see: https://academic.oup.com/plcell/article-abstract/33/4/1286/6119330).

Methods: Thylakoid isolation
"Thylakoids were isolated as described in (Hu et al., 2023) with minor modifications."
A direct citation to the original method for thylakoid isolation should be provided, in addition to the Hu et al. reference.

Section: Developmental priming of photoregulatory capacity in young leaves
This section reads as overly speculative. The authors are encouraged to revise it to more closely follow what the data can support, moderating the language where conclusions go beyond the experimental evidence.

Sentence clarity
"Comparisons between trees where shoots were 4 months, 7 months, and 1 year"
Please revise this sentence for grammatical clarity.

Abbreviation: LHCII
"Light complexes II (LHCII)"
Please correct the expansion of the abbreviation to 'Light Harvesting Complex II'.

Terminology: Fluctuating light
The term 'fluctuating light' is not precise in this context, as it typically implies a defined experimental protocol using alternating light intensities. The manuscript, however, describes plants grown under standard greenhouse conditions with supplemental lighting.
Fig. 1G
Please specify how the spectra shown were normalized.

Fig. 5A
Please clarify how samples were equalized prior to loading.

Fig. 5F and Fig. S10B
Please describe how band intensities were normalized between blots.

Fig. S6
Could the authors comment on the bands above lhcb1/b2 proteins that are located in the LHCII trimer band in the 2D electrophoresis of Arabidopsis samples? This is an unexpected outcome that warrants explanation.

Fig. S7
The authors should reconsider the application of Gaussian curve fitting to the data presented in this figure. The distributions shown (panels C & D) do not appear to follow a normal distribution, and fitting Gaussian curves to non-normal data is not statistically justified. An alternative fitting approach, or a more appropriate statistical description of the data, should be used.

Reviewer #4

(Remarks to the Author)
The manuscript entitled “Leaf development regulates 1 state transition capacity in trees” examines the regulatory mechanisms governing state transitions in trees. This process facilitates the fine-tuning of light-harvesting between Photosystem II and Photosystem I in plants (and other photosynthetic organisms). While extensively studied in the model organism Arabidopsis, information is scant for other species, particularly trees. Consequently, this study addresses a significant gap in our knowledge base. Overall, the experiments were carefully conducted, and the conclusions are supported by the data, with a few exceptions detailed below.

1. Given that the regulatory process of state transitions, as well as its capacity to respond to long-term acclimation, is strongly influenced by actual light intensity and quality, additional information on light regimes is essential. For instance, in greenhouse measurements, what were the light intensities at the L1 and L4 leaf levels? The same question applies to field measurements. Without this information, it remains uncertain to draw definitive conclusions about the developmental dependencies of state transition, as observed changes could also be attributable to variations in light intensity. The authors should provide further evidence supporting the claim that the capacity for state transitions in trees is solely developmentally dependent and not influenced by light intensity or quality. For example, can data on light intensity or quality be incorporated?

2. The parameter qT approaches zero in L4 leaves, yet biochemical data indicate the presence of an LHCI-LHCII-PSII state-transition complex and significant phosphorylation of LHCII. It is therefore necessary to question the reliability of qT as a quantifier of state transition. In the 77 K fluorescence spectra, a transition to the S2 state still appears to occur. The authors should therefore consider the potential limitations of this method.

3. The literature provides extensive data regarding ultrastructural changes in thylakoid architecture induced by state transitions. The manuscript would benefit from a discussion of its ultrastructural findings in the context of existing knowledge of this phenomenon in Arabidopsis.

4. The notation 'n>=5' biological samples, indicated in the figure legend of Fig. 4 (and similarly in other figures), warrants clarification. What exactly does this number represent— the number of leaves, or the number of five trees? The plots display approximately five data points. Is this an average across multiple leaves per tree?

5. Is there a specific rationale behind the classification of the four leaf layers in greenhouse-grown trees (lines 93-95)?

6. In lines 47-48, it should be noted as 'light harvesting complex II (LHCII)', rather than 'light complex II'.

7. In Fig. 3, the plastoglobuli should be labeled for L4.

8. Regarding Fig. S7, the histograms illustrating grana/chloroplast characteristics for L1 reveal heterogeneity, with two distinct distributions, particularly for S2. As such, these should not be described as a single Gaussian distribution.

Version 1:

Reviewer comments:

Reviewer #1

(Remarks to the Author)
The reviewers have carefully addressed my concerns and now given the required details and provided additional discussion. I find this version of the manuscript acceptable for publication.

Reviewer #3

(Remarks to the Author)

The authors have substantially revised the manuscript in response to my comments, and I am satisfied with all the responses, additional experiments, and corrections made. I thank the authors for carefully considering my suggestions and for the effort put into addressing them. The manuscript is now considerably clearer and the claims are better supported by the data.

Reviewer #4

(Remarks to the Author)
The authors addressed all of my concerns in an adequate manner.

Open Access This Peer Review File is licensed under a Creative Commons Attribution 4.0 International License, which permits use, sharing, adaptation, distribution and reproduction in any medium or format, as long as you give appropriate credit to the original author(s) and the source, provide a link to the Creative Commons license, and indicate if changes were made.
In cases where reviewers are anonymous, credit should be given to 'Anonymous Referee' and the source.
The images or other third party material in this Peer Review File are included in the article's Creative Commons license, unless indicated otherwise in a credit line to the material. If material is not included in the article's Creative Commons license and your intended use is not permitted by statutory regulation or exceeds the permitted use, you will need to obtain permission directly from the copyright holder.
To view a copy of this license, visit https://creativecommons.org/licenses/by/4.0/

REVIEWER COMMENTS

Reviewer #1 (Remarks to the Author):

"Leaf development regulates 1 state transition capacity in trees" is an interesting study that investigates state transitions in aspen leaves of different age, and compares a wild type hybrid aspen with its Stn7 KO that lacks state transitions. Three aspects make it specifically interesting: 1. Measurements on trees instead of Arabidopsis thaliana. 2. Aspect of leaf age is addressed. 3. Stn7 mutant is generated in aspen, second species after Arabidopsis. As trees are far more important for carbon fixation than Arabidopsis, this is a relevant topic to study. The work is original and combines greenhouse measurements with outdoor measurements, which make the relevance larger.

The manuscript could strongly benefit from more clearly explaining what has been done. In the current form the reader lacks some information to judge and interpret the data. Below I address, in the order of the manuscript, the points that I think need improvement. Minor issues are mixed with larger points.

We thank Reviewer for the careful reading of our manuscript and for the constructive suggestions. We appreciate the reviewer's positive assessment of the study and the helpful comments that improved the clarity and the presentation of the work. We have revised the manuscript accordingly and provide detailed responses to each comment below.

1.1 *Line 8 "photosystem II and I" you seem to follow the order of LET, but for the balance this does not matter, I suggest to write photosystem I and II.*

We agree and have revised the terminology throughout the manuscript from "photosystem II and I" to "photosystem I and II" for consistency.

1.2 Line 18 "reduced light-use efficiency in young leaves," you measure the PSII operating efficiency, while some readers might interpreted this as light-use efficiency a more common assumption might be CO_2 assimilation per photon.

We agree that the term "light-use efficiency" could be interpreted more broadly as carbon assimilation efficiency per absorbed photon. We have therefore revised the wording "light-use efficiency" to more precisely as "PSII operating efficiency".

1.3 Line 18-19 "slower growth under fluctuating but not constant light. These findings indicate that ST is important for performance under dynamic light conditions" The light intensity fluctuations are not the only difference between the greenhouse and the climate room, the light spectrum and changes therein are also different.

We agree that, in addition to differences in light intensity fluctuations, the greenhouse and climate room conditions also differed in light spectrum and changes. We have therefore revised the text to avoid attributing the observed growth differences solely to fluctuating light intensity.

1.4 Line 37 "on electron transport chain" -> of the electron transport chain

We have corrected the wording accordingly.

1.5 Line 99-100: "Although L1 leaves obviously had not yet a fully developed photosynthetic apparatus they were photosynthetically very active" why is this obvious? It looks like they have all photosynthetic components required? If you consider that L1 leaves have a lower Chl content, than the assimilation per absorbed photon might even be higher than for L2 leaves.

We agree that the original wording was too strong and potentially misleading. Our intention was to indicate that L1 leaves are still young and displayed characteristics of developing leaves. We have therefore revised the text accordingly.

1.6 Line 107 "Comparisons between trees where shoots were 4 months, 7 months, and 1 year, grown" please explain for those who do not work with aspen what age the leaves have in this case. The L1 leaves are the newly formed leaves, which are always younger than the L4 leaves. But how old are L1 leaves in a 1 year grown aspen? And L2 leaves?

We have now clarified the experimental design in the Methods section. The 7-month and 1-year trees were generated by repeated regrowth following cutting of the main stem, allowing new shoots and canopies to develop from the remaining stem and root system. Consequently, each developmental series formed a newly developed canopy containing comparable L1-L4 leaf layers. Thus the leaf layers were defined according to their relative position and developmental stage across the different tree age treatments, whereas the age of the underlying stem/root system differed. L1 corresponded to the uppermost fully expanded young leaves, L2 and L3 represented progressively older mature leaves, and L4 corresponded to the oldest fully expanded leaves.

1.7 Fig. 1 is this data from 4 months old trees?

The data shown in Fig.1 were obtained from 7-month-old-trees. We have now clarified this in the figure legend. Similar vertical trends in state transition capacity were also observed in trees of different ages and are shown in Fig. S4.

1.8 Throughout the manuscript T89 vs Stn7 is used, but Stn7 is generated from the T89. I think it would be better to say WT (T89) vs Stn7.

We agree and have revised the terminology throughout the manuscript from "T89" to "WT(T89)".

1.9 Fig. 1E in the M&M I can find the assimilation is measured at 150 uE, but I cannot find the light spectrum that was used, e.g. red/blue ratio preferably with wavelengths. Please add.

We have now added information regarding the spectral composition of the actinic light used for gas exchange measurements in the Method section. Measurements were performed under red light with a peak wavelength at 632 nm.

1.10Fig 4. Would it be possible to provide temperature data in the SI? I don't know if this is recorded in a weather station? If the temperature in October was low, than this could also be a reason for reduced qT. This might be added to the discussion.

We have now included ambient temperature data corresponding to the outdoor measurement period in Fig. S9D and expanded the discussion accordingly. While seasonal temperature changes may contribute to qT regulation, the progressive decline in qT did not directly follow the seasonal temperature trend, as temperature increased from spring to summer before decreasing toward autumn. Thus, the observed qT reduction cannot be explained solely by temperature changes.

1.11For the outdoor aspen measurements, how were the leaves assessed? What location in the canopy? Was any spectral data recorded? Is it likely that the leaves became shade adapted? The levels of Stn7 decrease in A.thaliana under long-term state 1 conditions (Willlig, 2012 www.plantphysiol.org/cgi/doi/10.1104/pp.111.187328). Maybe something similar happens in aspen?

We thank the reviewer for this this important comment. We have now clarified the canopy sampling strategy in the Methods section. For outdoor measurements, leaves were sampled from the outer protruding region of the lower canopy to minimize the local shading effects, and measurements were performed on randomly selected leaves withing the same canopy region.

We agree that long-term acclimation to canopy light conditions could potentially influence state transition capacity. Unfortunately, we didn't record the outdoor spectra following the season. But to evaluate if the spectra and intensity of the light could explain the reduced qT, we performed an additional shading experiment in which short aspens were grown beneath a dense canopy formed by taller aspens (new Fig.S11). Fluorescence emission spectra confirmed that the shaded plants experienced a more far-red enriched light environment compared to control plants. However, 12 days of shading did not reduce qT, suggesting shading alone is insurffient to explain the reduced qT observed in mature lower leaves.

We also agree that long-term state 1 conditions may potentially affect STN7 abundance, as previously reported in Arabidopsis (Willig et al., 2012). We therefore attempted to examine STN7 protein abundance in populus using the Agrisera STN7 antibody (AS164098). However, no specific signal was detected. After consultation with Agrisera, we confirmed that the 15-amino-acid peptide sequence used to generate the Arabidopsis STN7 antibody is not well conserved in Populus. We have therefore added this possibility to the Discussion as a potential mechanism that may contribute to the observed reduction in ST capacity in older leaves.

1.12 Line 195-196 "Also, 196 the BN-PAGE results (Fig. 2A) were not significantly different." In the example shown there is far less PSI-LHCII band relative to the PSI band in L4. What is the definition of "significant" used here?

We agree that the representative BN-PAGE image indicates a lower relative abundance of the PSI-LHCI-LHCII complex in L4 leaves compared with L1 leaves. The original

wording "not significantly different "was misleading and has been revised. To better quantify the observed differences, we performed densiometric analysis of the BN-PAGE bands using Image J. The relative proportion of PSI-LHCI-LHCII (PSI-LHCI-LHCII/(PSI-LHCI-LHCII + PSI-LHCI)) decreased from approx. 44% in L1 to 21% in L4, consistent with the partial reduction in LHCII phosphorylation observed in L4 leaves. The quantification has now been included in Tab. S2 and corresponding text has been revised accordingly.

1.13 Fig. 5C what is the colour/spectrum of light used to measure Phi(PSII)?

We have now clarified in the method section that Phi (PSII) was measured with red light with a peak wavelength at 632 nm.

1.14 For 5E it is mentioned after 4 months growth in greenhouse, are the other measurements made on the same plants? Measuring tree height is non-invasive, but is there also dry-weight or fresh weight data collected? The shape of the canopy could be changed.

We have now clarified in the manuscript that the height measurements shown in Fig. 5E were performed using 4-month-old plants, whereas the other physiological and biochemical analyses were conducted using 7-mont-old trees (in Method section). The 4-month-old plants were used for the height measurements to allow direct comparison between greenhouse-grown and platform-grown plants at the same developmental stage. Unfortunately, dry-weight and fresh-weight measurements were not collected in this experiment but we observed no obvious differences in overall pant architecture were observed between WT and stn7 plants during growth.

1.15 Line 226 use abbreviation for PSI and PSII.

We have revised the text to use the abbreviations PSI and PSII consistently.

1.16 Line 250 "for example, if there is a bottleneck at the PSII side rather than" please explain what you mean, e.g. if PSI is over-excited rather than PSII?

We agree that the original wording was unclear. Here, we intended to emphasize that room-temperature fluorescence measurements are dominated by PSII signals and therefore may not always reflect the extent of LHCII migration of association with PSI under all conditions. We have revised the sentence accordingly.

1.17 Line 316 "photoperiod Plants" -> photoperiod. Plants

Corrected as suggested.

1.18 Line 326: how was the canopy region defined?

We have now clarified the canopy sampling strategy in the Method section. For each tree, leaves were sampled from the outer protruding region of the lower canopy to minimize shading effects. Measurements were performed on randomly selected leaves from the same canopy region.

1.19Line 330: was qT only quantified from the Dual-PAM-100 measurements, or also from the ChloroSpec?

We apologize for the lack of the clarity. qT was quantified using both Dual-PAM-100 and ChloroSpec measurements. In the Dual-PAM-100 measurements, qT was calculated from integrated fluorescence values, whereas in the ChloroSpec measurments, qT was calculated based on the area of the fluorescence emission spectra. We have now clarified this in the method section.

1.20 Line 332-333: It reads like the light conditions lasted for 15 minutes, but it is 25 minutes, please indicated that the saturating flashes occur after the 15 minutes of continuous illumination. Please provide information on the durations and intensity of the saturating flash.

We thank the reviewer for pointing this out. We have clarified that leaves were illuminated with weak red light for 15 min to induce state 2, after which saturating flashes were applied over an additional 10 min period (3 flashes at 5 min intervals). We also added the duration and intensity of the saturating pulses to the method section.

1.21 Line 335: Vaygan is not the primary source for the equation and please provide the equation.

We agree and have now included the equation used to calculate state transitions directly in the method section and changed a more appropriate source.

1.22 Line 3412: please provide the formula for Phi(PSII) that was used and mention the light intensity also here.

We have now included the equation and used for Phi(PSII) calculation and the actinic light intensity used during the measurements.

1.23 396 TEM methods. Duration of fixation should be mentioned, as well as further protocol to embed and stain the sample. Or refer to a published protocol. It should also be mentioned how many different leaves were investigated, one can easily take 26 chloroplast from one leave, but that is only 1 biological replication.

The TEM methodology has now been revised to include a reference to the published protocol used for fixation and further procedures. We have also clarified the number of biological replicates by specifying the number of independent leaves analyzed in the revised manuscript.

1.24 SI

Fig. S2 "showing fluorescence traces during state 1 to state 2 induction." First state 2 is induced and next state 1.

The legend has been corrected to indicated that state 2 was induced first, followed by state 1.

1.25 Fig. 2B right the last two saturating pulses appear very high, how can this be?

We thank the reviewer for noticing this feature. We contacted WALZ technical support regarding the apparent discrepancy between the slow kinetics fluorescence trace and the FM' peaks during the saturating pulses. According to the WALZ engineer, this effect was caused by graphical limitations (conditional) affecting the signal resolution around saturating pulses in the slow kinetics recording, rather than by the fluorescence measurement itself. Following their recommendations, we adjusted the display resolution settings and obtained a graph in which the traces are fully consistent. The revised figure has been updated accordingly.

1.26 Fig. S8 what is the bin-size in the histogram? I count 7-8 bind per 10 membrane layers/grana, indicating that one bin is ~1.3 membrane. On the other hand the 3 data points are shown one after the other indicating bins of 3-4? Please make clear what the bins are. You can e.g. use transparent filling, to show the data on the same bins, or have the three bars together at the bin and have white space in between.

We thank the reviewer for this helpful suggestion. We have revised the histogram presentation to clearly indicate the bin width (2 membrane layers per bin) and modified the graphical representation by using overlapping transparent histograms with identical binning, allowing easier comparison of the distributions.

1.27 Fig. S10B please show an example of the blots. It is not clear how LHCII-p/LHCII was obtained, as LHCII is composed of Lhcb1-3. Was total LHCII estimated from the SDS-PAGE?

We thank the reviewer for pointing this out. LHCII-p/LHCII was estimated from immunoblot analyses using antibodies against Lhcb1, Lhcb2, phosphorylated Lhcb1 (Lhcb1-p), and phosphorylated Lhcb2 (Lhcb2-p). Representative immunoblots are shown in Fig.5, and we have clarified this in the figure legend.

1.28 The PsaA/CP43, the Chl a/b ratio and the thylakoid organisation indicate strong adaptation to shade light in L4. This is could be discussed more in the Discussion. It could be that adaptation to shade light provides a more precise explanation of the results than leaf development in general.

We agree that several characteristics observed in L4 leaves, including altered PSI/PSII stoichiometry, lower Chl a/b ratio, and enhanced thylakoid stacking, meet the shade acclimation traits. To better discuss it, we have performed an additional shading experiment in which short aspens were grown beneath a dense canopy formed by taller aspens (Fig. S11). Although the shaded plants experienced a far-red enriched light environment (Fig. S11B), 12 days of shading did not reduce qT in either top or bottom leaves compared to control plants (Fig. S11C). These results suggest that shading (around 2 weeks) alone is insufficient to explain the reduced qT in mature lower leaves and support the conclusion that developmental factor plays a dominant role in determining qT in this system. We have incorporated this new experiment and discussion into the revised manuscript.

Reviewer #2 (Remarks to the Author):

State transition (ST) is an important mechanism of adaptation to changing light conditions in photosynthetic organisms which has been mostly studied in Arabidopsis and in the unicellular alga Chlamydomonas. In this study the authors have examined ST in the tree model species aspen. They have quantified ST across vertical leaf layers of aspen subjected to long-term fluctuating light. Moreover they have also generated by CRISPR/Cas9 mutants deficient in ST to functionally test the role of ST in trees. Finally the authors have examined seasonal changes of ST in mature aspens and three other tree species grown under natural field conditions. The major conclusions of this study, based on physiological, biochemical, and ultrastructural analysis, is that in trees the leaf developmental stage impacts ST more than changing light conditions although it is not clear how this developmental control operates. This study also shows that in trees, the loss of the STN7 protein kinase has similar effects as in Arabidopsis, but surprisingly that in older leaves there is still appreciable LHCII phosphorylation with little ST. Overall, these are valuable results which extend our knowledge on the dynamic photoregulation of thylakoid architecture in angiosperms beyond Arabidopsis.

Although the technical quality of this study is high and the results are interesting, in my opinion it does not provide sufficient novel insights into the regulatory mechanisms of ST to justify publication in Nature Communications. I suggest that the authors submit their manuscript to a more specialized plant journal.

We thank the reviewer for the careful evaluation of our work and for recognizing both the technical quality of the study and the value of extending state-transition research beyond Arabidopsis. We appreciate the reviewer's concern that the original manuscript did not provide sufficient novel insights into the regulatory mechanisms underlying state transitions.

In response, we have substantially revised the manuscript and added several new experiments that strengthen both the mechanistic interpretation and the broader significance of the study. An important focus of the revised manuscript is an unexpected observation highlighted by the reviewer: in older leaves, fluorescence-derived state-transition capacity (qT) becomes almost undetectable, whereas substantial LHCII phosphorylation and PSI-LHCI-LHCII complex formation are still maintained. This finding indicates that different experimental approaches commonly used to assess state transitions can become uncoupled during leaf development.

To address this observation, we have revised the manuscript to distinguish more clearly between fluorescence-derived qT measurements, biochemical detection of PSI-LHCI-LHCII complexes, and LHCII phosphorylation. Since qT primarily reflects changes in the effective PSII antenna cross-section, whereas PSI-LHCI-LHCII formation reflects antenna association on the PSI side, our results suggest that PSI- and PSII-related manifestations of state transitions can become partially uncoupled during leaf development. This observation is not readily explained by the classical view of state

transitions as a fully coordinated redistribution process and provides a new perspective on how state transitions are functionally expressed in mature leaves. We have expanded the Discussion to examine possible mechanisms underlying this uncoupling, including developmental remodeling of the thylakoid architecture and changes in photosynthetic complex organization.

To further strengthen our conclusions, we performed several additional experiments. Firs, we carried out a canopy-shading experiment (new Fig. S11) to directly test whether the reduced qT observed in lower leaves could be explained by long-term exposure to a low-light, far-red-enriched environment. Although shaded plants experienced substantial changes in light quantify and quality, qT was not significantly reduced, indicating that light environment alone is insufficient to explain the developmental decline in state-transitions capacity. Second, we extended our analysis to Arabidopsis using whole-rosette chlorophyll fluorescence imaging (new Fig. S13). While most Arabidopsis leaves displayed similar qT values, the oldest leaves showed a slightly reduction in state transition capacity. These results suggest that developmental regulation of state transitions may represent a broader feature of higher plants, although it appears considerably more pronounce in trees, of which leaves undergo much longer developmental and structural maturation.

Together, these additions substantially strengthen the mechanistic framework of the study and broaden its physiological significance. Beyond establishing state transition regulation in trees, the revised manuscript provides new insight into how leaf development influences the functional expression of state transitions and reveals an unexpected uncoupling between PSII-based and PSI-based indicators of state-transitions activity. We therefore hope that reviewer and editor will consider that the revised manuscript now provides a broader conceptual advance regarding the developmental regulation of photosynthetic acclimation in higher plants.

Reviewer #3 (Remarks to the Author):

The manuscript investigates state transitions across leaf developmental stages in hybrid aspen and other tree species. The study addresses an interesting biological question and presents a valuable dataset; however, several aspects of the work would benefit from strengthening before the manuscript is ready for publication, including additional validation experiments that appear feasible within the existing framework and a more thorough engagement with the current mechanistic literature on state transitions.

We thank reviewer for the exceptionally careful and detailed evaluation of our manuscript. We appreciate the reviewer's many constructive comments regarding the interpretation of state transitions, terminology, experimental controls, and mechanistic discussion. In response, we have extensively revised the manuscript,

performed several new experiments and quantitative analyses. We believe these revisions have substantially improved the clarity and mechanistic depth of the manuscript. Our detailed responses are provided below.

Major Comments

3.1 Line 14

"Reduced ST correlated with increased grana stacking, but not with the LHCII phosphorylation, suggesting developmental remodeling may physically constrain LHCII redistribution."

This claim appears overly speculative given the data presented. The authors use qT as their primary measure of state transitions (ST) - qT is a parameter based on the changes of PSII fluorescence intensity in PSI- and PSII-specific light, and is related to the changes in PSII antenna cross section. It is important to note that an increase in qT does not necessarily imply long-distance relocation of LHCII from grana to stroma thylakoids; simple detachment of LHCII from the PSII core is sufficient to produce an increase in qT. The statement should therefore be revised to reflect what the data can and cannot support, and speculative language about the physical constraint of LHCII redistribution should either be substantiated or toned down accordingly.

3.2 Line 47

"Under PSII-light, the plastoquinone pool is reduced and the major light complexes II (LHCII) are phosphorylated by the chloroplastic serine/threonine-protein kinase STN7, and subsequently relocate from PSII to PSI, increasing the PSI antenna size (Cutolo et al., 2023; Jarvi et al., 2011; Longoni et al., 2019; Schiphorst et al., 2022; Wunder et al., 2013). This state is called state 2 (Bellafiore et al., 2005)."

This description of the state transitions mechanism is somewhat outdated, as it does not account for more recent evidence demonstrating only partial migration of LHCII between grana and stroma thylakoid compartments. Thorough analyses have indicated that long distance LHCII migration is considerably more limited than the classical model suggests. The authors should consider incorporating and discussing the revised perspective on this process, including, in particular, the work from Matt Johnson's laboratory.

3.3 Additionally, there is an internal terminological inconsistency that warrants attention. The definition given here for state 2 implies full LHCII relocation, yet throughout the manuscript, state 2 is operationally identified via qT measurements, which reflect a decrease in PSII antenna cross-section (i.e., LHCII detachment from PSII core) rather than confirming physical relocation. Similarly, the 77 K fluorescence emission data - cited as evidence for state transitions - reports on increased LHCII association with the PSI-LHCI complex, which is mechanistically distinct from qT. The authors are encouraged to be more precise and consistent in their use of terminology across the manuscript, clearly distinguishing between LHCII detachment and LHCII relocation where relevant.

3.1-3.3 We thank the reviewer for these insightful comments regarding the mechanistic interpretation and terminology about state transitions. We agree that qT

measurements primarily reflect changes in effective PSII antenna cross-section and do not by themselves demonstrate long-distance migration of LHCII between grana and stroma lamellae. We also agree that the classical model describing complete LHCII relocation oversimplifies the current understanding of state transitions.

In accordance with recent studies, particularly work from Matt Johnson's laboratory, we have revised the manuscript to describe qT association and disassociation of LHCII with PSII, rather than complete migration of antenna complexes. Throughout the revised manuscript, we now distinguish more carefully between qT measurements, PSI-LHCI-LHCII association observed by BN-PAGE and 77 K fluorescence measurements, and physical redistribution of LHCII complexes within thylakoid membranes. Accordingly, we have revised several statements throughout the manuscript to avoid overinterpreting qT as direct evidence for large-scale LHCII migration or physical redistribution. We also toned down the previous statement suggesting that developmental remodeling may physically constrain LHCII redistribution, and instead discuss our observations in terms of altered flexibility of antenna reorganization and changes in the relationship between fluorescence derived qT and biochemical signatures of ST in PSI side.

3.4 Line 73

"We demonstrate that state transition in trees is strongly dependent on the leaf developmental stage, which has a stronger effect than light environment alone."

The authors make a strong claim about the primacy of developmental stage over light environment, yet the experimental design does not include complementary experiments in Arabidopsis that would help contextualize this finding within the broader literature. Including data from Arabidopsis rosettes - where leaf position serves as a well-established proxy for leaf age - would provide a useful bridge between the extensive existing literature on Arabidopsis state transitions and the novel tree-focused work presented here. Specifically, PAM imaging-based qT measurements across leaves at different rosette positions could help disentangle whether the observed developmental decline in state transition capacity is a tree-specific phenomenon or a more general feature of angiosperm leaf maturation. This would considerably strengthen the conclusions of the paper.

Furthermore, the authors do not appear to have measured the light spectra actually reaching leaves at different canopy layers. Light filtered through upper leaves is spectrally enriched in far-red wavelengths, which could independently influence state transition induction. The authors should address whether the observed canopy gradient in state transitions might be partly attributable to differences in spectral composition rather than (or in addition to) leaf developmental stage.

We thank the reviewer for this valuable suggestion. We have now performed additional qT measurements on Arabidopsis rosette leaves using a SpeedZen fluorescence imaging system (new Fig. S12). To minimize the influence of leaf angle and self-shading withing the rosette, leaves were also separated into three developmental groups (young, mature, and old leaves), detached and measured on the same flat platform.

Similar to aspen, older Arabidopsis leaves generally displayed a slightly reduced qT values compared with younger and mature leaves, although the differences were less pronounced than those observed between L1 and L4 aspen leaves. One possible explanation is that the developmental differences between canopy layers in aspen are substantially larger, with leaves differing in age by several months, whereas Arabidopsis leaves mature much more rapidly and therefore exhibit a smaller developmental gradient withing a rosette.

Regarding the second point, we agree that differences in light intensity and spectral quality between canopy layers could potentially influence ST capacity. We have therefore performed a dedicated shading experiment (new Fig. S11). In this experiment, short aspens were grown beneath a dense canopy formed by taller aspens, generating a "more extreme" low-light and far-red enriched environment. The spectra were shown in Fig. S11. However, after 12 days of shading, qT in either top or bottom leaves do not differ significantly from the control plants. These results suggest that shading alone is insufficient to explain the strongly reduced qT in mature lower leaves and support that developmental factors play a dominant role in determining qT capacity in this system.

3.5 Line 123

"Under state 2, L1 thylakoids displayed PSI-LHCI-LHCII complexes, which were absent in state 1 (Fig. 2A). This complex was also present, although at a lower level, in L4 in state 2 (Fig. 2A)."

Before drawing conclusions from the BN-PAGE data, the authors should verify and report the efficiency of digitonin solubilization across the different variants. Specifically, was chlorophyll content measured in the pellet fraction following centrifugation after digitonin treatment? A comparison of this efficiency across leaf positions and state conditions is essential for valid interpretation. Additionally, a quantitative analysis of the BN-PAGE data - such as calculating the intensity of the PSI-LHCI-LHCII band relative to the PSI-LHCI band - would considerably strengthen the claim, as the overall lane intensities across the studied variants appear to differ, which complicates direct comparison of band patterns.

We thank the reviewer for these important suggestions. We have now quantified digitonin solubilization efficiency by measuring chlorophyll remaining in the pellet fraction after centrifugation following digitonin treatment. Solubilization efficiency was lower in L4 thylakoids (24+-4%) compared to L1 thylakoids (35+-1%) (new Fig.S6C), indicating differences in detergent extraction efficiency between leaf layers. The lower digitonin extraction efficiency observed in L4 is consistent with the reduced proportion of stromal lamellae and increased grana stacking in TEM analysis. Importantly, despite the lower digitonin solubilization efficiency in L4 thylakoids, PSI-LHCI-LHCII complexes were still clearly detected in L4 state 2 samples, even though qT values were very low. This observation supports that the fluorescence-derived qT measurements (PSII side) and PSI-LHCI-LHCII formation (PSI side) can become partially uncoupled, as discussed in the revised manuscript.

We also performed densitometric analysis of the BN-PAGE bands using ImageJ to quantify the relative abundance of the PSI-LHCI-LHCII complex. The relative proportion of PSI-LHCI-LHCII in (PSI-LHCI-LHCII+PSI-LHCI) decreased from 44% in L1 to 21% in L4, consistent with the partial reduction in LHCII phosphorylation observed in L4 leaves. The quantification has now been included in Tab.S2 and the corresponding text has been revised accordingly.

3.6 Line 128

"Although the relative abundances of some individual proteins varied (Fig. 2C)."

No quantitative analysis is provided in the manuscript to support this statement. Additionally, please clarify how the samples loaded in each lane were equalized (e.g., on a chlorophyll, protein, or other basis).

The statement has been revised to avoid overinterpretation of the Coomassie-stained SDS-PAGE profiles. We have also clarified in the revised manuscript that equal amounts of chlorophyll were loaded in each lane prior to electrophoresis.

3.7 Line 147

"Additionally, plastoglobuli were observed in L4 leaves (Fig. 3B, Fig. S7). Plastoglobuli are associated with older aspen leaves and accumulate as the leaves enter senescence; however, they were less abundant in L4 than in fully senescing aspen leaves (Keskitalo et al., 2005)."

This statement and the subsequent description give the misleading impression that plastoglobules are exclusive to L4 leaves. However, as is clearly visible in the TEM images presented in the manuscript, plastoglobules are also present in younger leaves - albeit at smaller sizes. The developmental increase in plastoglobule size is a well-documented phenomenon in chloroplast biology, and plastoglobules are in fact a constitutive feature of plastids at all developmental stages, including etioplasts. The text should be revised to reflect this accurately. Furthermore, the authors should provide quantitative data on plastoglobule size (diameter) and abundance per chloroplast across the different leaf positions to support their comparative statements.

We agree with reviewer. The text has been revised to clarify that plastoglobuli were observed in chloroplasts from all developmental stages but became larger and more abundant in L4 leaves. We agree that plastoglobuli are constitutive components of plastids throughout development and should not be presented as exclusive features of older leaves. We additionally quantified plastoglobuli diameter and abundance per chloroplast in L1 and L4 leaves, and these data have now been incorporated into the revised manuscript and Fig. S7.

3.8 Fig. 3

The data presented here illustrate the well-known developmental progression of thylakoid network organization - an initial increase in grana diameter followed by a build-up of membrane layers - a pattern that has previously been described in crop plants and other species. However, no references are cited to acknowledge this prior literature. The authors should include appropriate citations documenting the developmental trajectory of thylakoid architecture to contextualize their findings.

We have now added references describing developmental remodeling of chloroplast ultrastructure and thylakoid organization during leaf maturation and aging in higher plants, including previous studies reporting increased grana stacking and changes in grana architecture with leaf development in other species.

3.9 Fig. 4 — Bottom panel: seasonal changes in state transition capacity

To ensure that the seasonal changes in qT reflect genuine variation in state transition capacity rather than shifts in the stoichiometry of the photosynthetic machinery, the authors should provide supporting data demonstrating that the ratio of PSII core complexes to LHCII antenna is stable across the sampling time points. Changes in the PSII/LHCII ratio could significantly influence qT values independently of any modulation of state transitions per se, and this potential confound should be addressed.

We agree that changes in photosystem stoichiometry could influence fluorescence-derived qT. To address this possibility, we performed additional immunoblot analysis on outdoor populus across the seasonal time course. The results showed a gradual decrease in the relative LHCII/PSII ratio during the season, and these data have now been added in Fig.S8. We have now incorporated this observation into the revised manuscript and now discuss that seasonal changes in photosystem stoichiometry may contribute to the observed reduction in qT by altering the effective PSII antenna size.

3.10 Line 244

"Our data indicate that the limitation of ST in older/lower leaves is not simply due to the absence of LHCII phosphorylation or PSI association (with LHCII). In L4 leaves, we still observed substantial LHCII phosphorylation (60% of T89) and PSI-LHCI-LHCII assemblies under state 2 conditions, yet chlorophyll fluorescence measurements showed only little ST."

While this is an intriguing observation, the authors do not offer a mechanistic hypothesis to explain the apparent disconnect between LHCII phosphorylation, PSI-LHCI-LHCII assembly, and the qT-based assessment of state transitions. A more thorough discussion of possible explanations would be valuable. Furthermore, as noted above, this apparent paradox may also partly reflect the definitional ambiguity between qT (which reports on LHCII detachment from PSII) and state transitions as defined by LHCII association with PSI. A more careful treatment of these distinctions in the discussion would help the reader interpret the data appropriately.

We agree that the apparent disconnection between sustained LHCII phosphorylation, detectable PSI-LHCI-LHCII assemblies, and strongly reduced qT in older leaves requires further mechanistic consideration. In the revised Discussion, we have therefore expanded this section. We now discuss that qT primarily reflects changes in the effective PSII antenna cross-section rather than directly measuring large-scale physical redistribution of LHCII within the thylakoid membrane. We further discuss two possible factors that may contribute to the reduced qT observed in older leaves despite sustained LHCII phosphorylation. 1. Developmental reduction in the relative LHCII/PSII ratio. 2. Increased grana stacking, which may stabilize PSII-LHCII connectivity and limit the flexibility of antenna reorganization.

3.11 Line 289

"As it has been suggested that the STN7 kinase may have other roles (Poudyal et al., 2020) in addition to regulating state transitions, the question is if the lack of ST or some other STN7 functions is causing this."

This sentence is unclear and would benefit from revision.

We have revised the text to clarify that STN7 has been included not only in classical state transitions but also in the regulation thylakoid remodeling and photosynthetic complex stability through CP29 phosphorylation during leaf aging.

3.12 Line 299

"This decline occurs despite sustained LHCII phosphorylation, suggesting that the functional extent of state transitions is influenced by the structural maturation of the thylakoids."

This statement is problematic for reasons discussed above: if state transitions are operationally defined throughout this manuscript by qT measurements - which reflect changes in PSII antenna cross-section rather than direct structural rearrangements - then invoking grana metrics as an explanatory variable requires careful justification. The authors should either provide a mechanistic link between grana structure and qT or qualify their interpretation accordingly.

We agree that qT measurements do not directly report large-scale structural rearrangements within the thylakoid membrane. In the revised manuscript, we have therefore clarified that qT primarily reflects changes in the effective PSII antenna cross-section rather than direct physical migration of LHCII. We have additionally softened our interpretation regarding grana structure and now discuss increased grana stacking as a potential structural factor that may constrain the flexibility of antenna reorganization reflected in qT measurements, rather than as direct evidence for altered ST per se. In addition, we expanded the discussion section to distinguish more clearly between fluorescence-derived qT measurements and biochemical detection of PSI-

LHCI-LHCII assemblies, consistent with recent models proposing dynamic and partial redistribution of LHCII during state transitions.

Minor Comments

Abstract

3.13 "reports on the regulation and ecological significance of ST in other angiosperms remain largely unknown"

This sentence implies that the present work will comprehensively address the ecological significance of state transitions, which it does not. The abstract should be revised to more accurately reflect the scope of the study.

We agree that the original wording overstated the scope of the study. We have therefore revised the Abstract to refer to the "physiological significance" rather than the "ecological significance" of ST in other angiosperms.

3.14 Terminology: FV/FM

"maximum PSII quantum yield (FV/FM)"

This notation is outdated. The authors should use the updated nomenclature as recommended in the recent guidelines (see: https://academic.oup.com/plcell/article-abstract/33/4/1286/6119330).

We have revised the manuscript according to the updated chlorophyll fluorescence nomenclature guidelines throughout the text, figures and legends.

3.15 Methods: Thylakoid isolation

"Thylakoids were isolated as described in (Hu et al., 2023) with minor modifications."

A direct citation to the original method for thylakoid isolation should be provided, in addition to the Hu et al. reference.

We agree and have revised to include a direct citation to the original thylakoid isolation protocol described by Caffarri et al., (2009).

3.16 Section: Developmental priming of photoregulatory capacity in young leaves

This section reads as overly speculative. The authors are encouraged to revise it to more closely follow what the data can support, moderating the language where conclusions go beyond the experimental evidence.

We have revised and moderated the Discussion throughout this section to more closely align with the experimental evidence. In particular, we softened interpretations regarding developmental optimization and the relationship between thylakoid structure and qT, clarified the limitations of fluorescence-derived qT measurements, and reframed several conclusions as possible explanations rather than definitive mechanisms.

3.17 Sentence clarity

"Comparisons between trees where shoots were 4 months, 7 months, and 1 year"

Please revise this sentence for grammatical clarity.

We agree and have revised the sentence for improved grammatical clarity.

3.18 Abbreviation: LHCII

"Light complexes II (LHCII)"

Please correct the expansion of the abbreviation to 'Light Harvesting Complex II'.

We have corrected the abbreviation.

3.19 Terminology: Fluctuating light

The term 'fluctuating light' is not precise in this context, as it typically implies a defined experimental protocol using alternating light intensities. The manuscript, however, describes plants grown under standard greenhouse conditions with supplemental lighting.

We agree that the term "fluctuating light" may imply a defined experimental light-shift protocol. We have therefore revised the manuscript to use more precise terminology such as "dynamic greenhouse light conditions" or "naturally variable light conditions". Nevertheless, unlike the climate room, the greenhouse environment was not constant, as natural sunlight varied continuously, and supplemental light was automatically regulated depending on ambient irradiance to maintain approximately 150 µE. Thus, plants experienced a dynamically changing light environment rather than strictly constant illumination.

3.20 Fig. 1G

Please specify how the spectra shown were normalized.

We have revised the figure legend to specify that the spectra shown in Fig. 1G were normalized at 692 nm.

3.21 Fig. 5A

Please clarify how samples were equalized prior to loading.

We have revised the figure legend to clarify that samples were equalized on chlorophyll content prior to loading.

3.22 Fig. 5F and Fig. S10B

Please describe how band intensities were normalized between blots.

For comparison between blots, the L1 WT (T89) state 2 sample was included as a reference and used for normalization in the quantitative analysis. This information has now been added .

3.23 Fig. S6

Could the authors comment on the bands above lhcb1/b2 proteins that are located in the LHCII trimer band in the 2D electrophoresis of Arabidopsis samples? This is an unexpected outcome that warrants explanation.

We agree that the Arabidopsis "LHCII trimer" region contains additional proteins besides the major LHCII components. These bands likely represent PSII-associated proteins or partially dissociated PSII subcomplexes co-migration with the LHCII-containing region during BN-PAGE separation. Digitonin preferentially solubilizes stroma lamellae and grana margins, where PSII assembly and repair intermediates are enriched, which may contribute to this pattern. In our experiments, Arabidopsis thylakoids were more sensitive to digitonin solubilization (with 2% at RT) than aspen thylakoids, likely resulting in more extensive membrane disassembly and co-migration of PSII-related proteins, to avoid overinterpretation, we have revised the figure labeling form "LHCII trimer" to "LHCII trimer/partially disassembled PSII"

3.24 Fig. S7

The authors should reconsider the application of Gaussian curve fitting to the data presented in this figure. The distributions shown (panels C & D) do not appear to follow a normal distribution, and fitting Gaussian curves to non-normal data is not statistically justified. An alternative fitting approach, or a more appropriate statistical description of the data, should be used.

We agree that Gaussian fitting was not the most appropriate approach for these distributions. We have therefore revised Fig. S7 by replacing the Gaussian fitting curves with kernel density smoothing, which provides a non-parametric representation of the data distribution without assuming normality.

Reviewer #4 (Remarks to the Author):

The manuscript entitled "Leaf development regulates 1 state transition capacity in trees" examines the regulatory mechanisms governing state transitions in trees. This process facilitates the fine-tuning of light-harvesting between Photosystem II and Photosystem I in plants (and other photosynthetic organisms). While extensively studied in the model organism Arabidopsis, information is scant for other species, particularly trees. Consequently, this study addresses a significant gap in our knowledge base. Overall, the experiments were carefully conducted, and the conclusions are supported by the data, with a few exceptions detailed below.

We thank reviewer for the positive evaluation of our study and for recognizing the importance of investigating state transitions in trees. We appreciate the reviewer's constructive suggestions regarding environmental effects, methodological clarifications, and interpretation of the physiological data. Detailed responses to each comment are provided below.

4.1. Given that the regulatory process of state transitions, as well as its capacity to respond to long-term acclimation, is strongly influenced by actual light intensity and quality, additional information on light regimes is essential. For instance, in greenhouse measurements, what were the light intensities at the L1 and L4 leaf levels? The same question applies to field measurements. Without this information, it remains uncertain to draw definitive conclusions about the developmental dependencies of state transition, as observed changes could also be attributable to variations in light intensity. The authors should provide further evidence supporting the claim that the capacity for state transitions in trees is solely developmentally dependent and not influenced by light intensity or quality. For example, can data on light intensity or quality be incorporated?

We fully agree that claiming that state transition capacity in trees is mainly developmentally dependent requires additional supporting evidence, since both the light intensity and the spectral quality reaching L1 and L4 leaves are different. We therefore performed an additional shading experiment. Short aspens were divided into two groups: one group was grown without shading used as a control, while the second group was grown beneath a dense canopy (in L4) formed by taller aspens (Fig. S11). Although the shaded plants experienced a far-red enriched (spectra were shown) and low-light intensity environment, 12 days of shading did not reduce qT in either top or bottom leaves compared to control plants.

These results suggest that shading alone is insufficient to explain the reduced qT in mature lower leaves and support the conclusion that developmental factor play a (more) dominant role in determining qT in this system. We have incorporated this new experiment and related discussion into the revised manuscript.

4.2. The parameter qT approaches zero in L4 leaves, yet biochemical data indicate the presence of an LHCI-LHCII-PSII state-transition complex and significant phosphorylation of LHCII. It is therefore necessary to question the reliability of qT as a quantifier of state transition. In the 77 K fluorescence spectra, a transition to the S2 state still appears to occur. The authors should therefore consider the potential limitations of this method.

We agree that fluorescence-derived qT measurements do not fully capture all aspects of state transition-related antenna reorganization. We have therefore revised the Discussion to clarify that qT primarily reflects changes in the effective PSII antenna cross-section measured by chlorophyll fluorescence at RT rather than directly measuring large-scale physical redistribution of LHCII between two PSs. We further discuss that PSI-LHCI-LHCII assemblies and LHCII phosphorylation were still observed in L4 leaves despite almost "0" qT. These observations suggest that fluorescence-derived qT measurements and PSI-associated LHCII assemblies report partially distinct aspects of ST.

4.3. The literature provides extensive data regarding ultrastructural changes in thylakoid architecture induced by state transitions. The manuscript would benefit from a discussion of its ultrastructural findings in the context of existing knowledge of this phenomenon in Arabidopsis.

We have expanded the Discussion to place our ultrastructural observations in the context of previous Arabidopsis studies on thylakoid remodeling during state transitions. In particular, we now discuss earlier reports showing that LHCII phosphorylation and state transitions are associated with dynamic changes in grana organization and thylakoid stacking (Chuartzman et al., 2008; Hepworth et al., 2021; Wood et al., 2018), and relate these findings to the developmental increase in grana stacking observed in mature aspen leaves.

4.4. The notation 'n>=5' biological samples, indicated in the figure legend of Fig. 4 (and similarly in other figures), warrants clarification. What exactly does this number represent— the number of leaves, or the number of five trees? The plots display approximately five data points. Is this an average across multiple leaves per tree?

We have revised the figure legends to clarify that n refers to the number of individual leaves measured. Leaves were collected from five independent trees, and each data point represents the qT value measured from a single leaf.

4.5. Is there a specific rationale behind the classification of the four leaf layers in greenhouse-grown trees (lines 93-95)?

We have revised the Methods section to clarify the rationale for the leaf layer classification. The L1-L4 layers were defined according to their relative vertical

positions within the canopy, ranging from the uppermost fully expanded leaves (L1) to the lower most mature leaves (L4).

4.6. In lines 47-48, it should be noted as 'light harvesting complex II (LHCII)', rather than 'light complex II'.

We have now corrected the text to "light-harvesting complex II (LHCII)" in the manuscript.

4.7. In Fig. 3, the plastoglobuli should be labeled for L4.

Plastoglobuli in the L4 chloroplast image are now indicated with orange triangles in the revised Fig. 3 legend and image annotations.

4.8. Regarding Fig. S7, the histograms illustrating grana/chloroplast characteristics for L1 reveal heterogeneity, with two distinct distributions, particularly for S2. As such, these should not be described as a single Gaussian distribution.

We agree with the reviewer that the distributions are heterogeneous and are not adequately described by a single Gaussian distribution. We have therefore removed the Gaussian fitting and replaced it with non-parametric kernel density smoothing in the revised Fig. S7.

*** See Nature Portfolio's author and referees' website at www.nature.com/authors for information about policies, services and author benefits.*

This email has been sent through the Springer Nature Tracking System NY-610A-NPG&MTS

Confidentiality Statement:

This e-mail is confidential and subject to copyright. Any unauthorised use or disclosure of its contents is prohibited. If you have received this email in error please notify our Manuscript Tracking System Helpdesk team at http://platformsupport.nature.com .

Details of the confidentiality and pre-publicity policy may be found here http://www.nature.com/authors/policies/confidentiality.html

Privacy Policy | Update Profile
